# Blocking distinct interactions between Glioblastoma cells and their tissue microenvironment: A novel multi-targeted therapeutic approach

**DOI:** 10.1038/s41598-018-23592-z

**Published:** 2018-04-03

**Authors:** Melanie Mettang, Viola Meyer-Pannwitt, Georg Karpel-Massler, Shaoxia Zhou, Neil O. Carragher, Karl Josef Föhr, Bernd Baumann, Lisa Nonnenmacher, Stefanie Enzenmüller, Meike Dahlhaus, Markus D. Siegelin, Sebastien Stroh, Daniel Mertens, Pamela Fischer-Posovszky, E. Marion Schneider, Marc-Eric Halatsch, Klaus-Michael Debatin, Mike-Andrew Westhoff

**Affiliations:** 1 Department of Pediatrics and Adolescent Medicine, University Medical Center Ulm, Ulm, Germany; 2 Institute of Physiological Chemistry, University Medical Center Ulm, Ulm, Germany; 3 Department of Internal Medicine III, University Medical Center Ulm, Ulm, Germany; 4 Mechanisms of Leukemogenesis, German Cancer Research Center (DKFZ), Heidelberg, Germany; 5 Department of Neurosurgery, University Medical Center Ulm, Ulm, Germany; 6 Department of Clinical Chemistry, University Medical Center Ulm, Ulm, Germany; 7 Edinburgh Cancer Research Center UK, Institute of Genetics and Molecular Medicine, University of Edinburgh, Edinburgh, United Kingdom; 8 Department of Anesthesiology, University Medical Center Ulm, Ulm, Germany; 9 Department of Pathology and Cell Biology, Columbia University Medical Center, New York, NY USA; 10 Department of Neurology, University Medical Center Ulm, Ulm, Germany

## Abstract

Due to the highly invasive nature of Glioblastoma (GB), complete surgical resection is not feasible, while motile tumour cells are often associated with several specific brain structures that enhance treatment-resistance. Here, we investigate the therapeutic potential of Disulfiram and Carbenoxolone, that inhibit two distinct interactions between GB and the brain tissue microenvironment: stress-induced cell-matrix adhesion and gap junction mediated cell-cell communication, respectively. Increase in cell numbers of tumour-initiating cells, which are cultured in suspension as cell clusters, and adherent differentiated cells can be blocked to a similar extent by Carbenoxolone, as both cell populations form gap junctions, but the adherent differentiated cells are much more sensitive to Disulfiram treatment, which – via modulation of NF-κB signalling – interferes with cell-substrate adhesion. Interestingly, inducing adhesion in tumour-initiating cells without differentiating them does not sensitize for Disulfiram. Importantly, combining Disulfiram, Carbenoxolone and the standard chemotherapeutic drug Temozolomide reduces tumour size in an orthotopic mouse model. Isolating GB cells from their direct environment within the brain represents an important addition to current therapeutic approaches. The blockage of cellular interactions via the clinically relevant substances Disulfiram and Carbenoxolone, has distinct effects on different cell populations within a tumour, potentially reducing motility and/or resistance to apoptosis.

## Introduction

Glioblastoma (GB), formerly Glioblastoma multiforme, is the most common cancer of the central nervous system with poor prognosis exemplified by patient survival of about one year after diagnosis^1^. Despite intensive treatment involving tumour resection, radiation and chemotherapeutic treatment with Temozolomide (TMZ), GB inevitably recurs^2^. GB is a highly aggressive malignancy with rather unique features: while it only rarely metastasizes outside the neuraxis^3^, it is almost invariably found to be highly invasive upon presentation^4^. It is still debated whether GB should be viewed as a tumour within the brain or a systemic whole brain disease. The latter view had been particularly popular among early radiation oncologists^5^ and is currently gaining favour once more^6^. In extreme cases, GB can be lethal in the complete absence of tumour bulk^4^. The unfocused nature of this disease makes localized treatment, e.g. maximal safe surgery, particularly ineffective^7^. After excision of the tumour bulk, recurrence manifests within 2–3 cm of the resection cavity in more than 95% of cases^4^. The invading GB cells often associate with distinct anatomic structures, e.g. myelinated axons, basement membranes of blood vessels, other basement membrane-like structures, and the so-called “secondary structures of Scherer”^8^. These structures are known to confer increased resistance to apoptosis^9,10^ by inducing various pro-survival signalling cascades – a phenomenon we have previously referred to as AMAR, or adhesion-mediated apoptosis resistance^11^. Previous targeted therapies blocking individual adhesion receptors such as cilengitide (inhibitor of αvβ3 and αvβ5 integrins) have had limited efficacy in GB clinical trials^12^. The poor efficacy of targeted adhesion blocking therapies may be limited in part by redundancy in multiple adhesion receptor mediated signalling events, which confer AMAR across the disseminated GB microenvironment of the brain. Therefore, a multi-targeted approach of blocking adhesion signalling in GB should minimize the interaction of tumour cells with their surroundings, reduce invasion and re-sensitize cancer cells for apoptosis. To test this hypothesis, we selected two forms of cellular interaction which have been shown to contribute to GB biology – cell-matrix interactions and gap junctions.

Cell-matrix interactions are usually formed via integrin engagement that tethers the cell to its surroundings and activates complex intracellular signalling cascades^11^. We recently showed that invasive GB cells are associated with fibronectin that is secreted and processed by the tumour cells via plasminogen and matrix metallopeptidases^13^. Importantly, the creation of this new extracellular matrix (ECM)-based microenvironment was initiated upon a stress response resulting from the reduction of cell-cell interactions, which triggered NF-κB activation^13^. Blocking NF-κB activation via the nonspecific, but well-tolerated, inhibitor Disulfiram (DIS) reduces both tumour bulk and cellular invasion in an orthotopic mouse model^13^. This is also in line with previous data that suggest that Disulfiram-mediated inhibition of NF-κB sensitizes colorectal cancer cells for cell death^14^.

In contrast, gap junctions are formed between adjacent cells. They have been described to form transiently during invasion between GB cells and astrocytes as well as part of long-distance multicellular network structures^15,16^. Our own data, using the glycyrrhetinic acid-derivative Carbenoxolone (CBX) for the inhibition of gap junctions, also suggest that stable gap junctions contribute to the close cell-cell interaction associated with the tumour bulk, and that these structures clearly contribute to apoptosis resistance^17^. Therefore, we postulated that inhibition of cell-fibronectin interaction should mainly affect invasive/stressed cells, while blocking gap junctions should influence both invasive cells and tumour bulk - the latter more strongly however, as gap junctions are more stable in that context.

To mimic the intratumoral heterogeneity, i.e. the existence of competing and supporting subpopulations of tumour cells that adds to the complexity of the disease^18^ in an experimental system, we used two genetically identical cell populations with different forms of dominant cellular interactions. GB stem-cell like cells, also referred to as tumour-initiating cells (TiCs), are cultured as free-floating clusters, i.e. communicating mainly via stable gap junctions. Within the tumour, they make up only a small fraction of total cells, but are responsible for the overall phenotype and are thought to be resistant to radio- and chemotherapy and thus lead to persistent tumour regrowth^19,20^. The main tumour bulk consists of differentiated GB cells (Difs), which experimentally are cultured adherently, forming cell-matrix contacts^13^. Importantly, functional differences between TiCs and Difs are not only due to different forms of cellular contacts, but also due to their alternative states of differentiation, even though they are derived from the same GB patient, i.e. of identical genetic origin.

In this work, we evaluated the potential of a complex combination therapy that is primarily aimed to isolate GB cells within the brain from their microenvironment. Specifically, we were able to show that the two major forms of cellular interactions, cell matrix-interaction and gap junctions, were blocked in primary GB cells via treatment with DIS and CBX. Importantly the two compounds did not antagonize each other when given in combination and when combined with chemotherapy, such as TMZ in an orthotopic mouse model.

## Methods

### Primary tumour material

After patients’ informed consent, GB-TiC spheres were grown from surgical specimens and cultured as previously described^21^. Briefly, TiCs were cultured in supplemented DMEM/F-12 (HAM) medium (Invitrogen, Darmstadt, Germany). Adherent TiCs were cultured in non-tissue culture flasks (Sarstedt, Nümbrecht, Germany) pre-treated with collagen type I (BD Biosciences, Bedford, MA, USA) for 1 hour at 37 °C. Dif cells were grown in supplemented DMEM medium (Invitrogen).

### Changes in cell number

Cells were counted using CASY1 DT (OMNI Life Science, Bremen, Germany), as previously described^13^.

### Apoptosis measurement

Apoptosis was determined by fluorescence-activated cell-sorting (FACScan, Becton Dickinson, Heidelberg, Germany) analysis, using DNA fragmentation of propidium iodide (PI)-stained nuclei as a surrogate readout^13^. Shown is the specific DNA fragmentation, calculated as follows: 100× (experimental DNA fragmentation (%) - spontaneous DNA fragmentation (%))/(100%-spontaneous fragmentation (%)) and analysed by two-way ANOVA followed by Bonferroni’s post-test.

### Inhibitors and drugs

Tetraethylthiuram disulfide (DIS), Temozolomide (TMZ) and Carbenoxolone disodium (CBX) were obtained from Sigma-Aldrich (Steinheim, Germany) and dissolved in DMSO (DIS and TMZ) or water (CBX). For *in vivo* experiments CBX was replaced by INI-0602 (Wako Pure Chemical Ind., Ltd., Japan).

### Time-lapse photography

Time-lapse photography was performed by taking a picture of the cells every ten minutes over a time frame of at least 48 hours under a CK40 microscope with a CAMEDIA C-4040ZOOM digital camera (Olympus, Hamburg, Germany). The relative speed of cells was quantified using ImageJ software (Rasband, W.S., ImageJ, U. S. National Institutes of Health, Bethesda, Maryland, USA, http://imagej.nih.gov/ij/, 1997–2011).

### Orthotopic mouse model

1.0 × 10^5^ G35-TiCs were transplanted into NOD.Cg-PrkdcscidII2rgtm1WjI/SzJ mouse brains as previously described^22^ and in accordance with relevant guidelines and regulations. Tissue samples were stained with haematoxylin and for expression of vimentin (Abcam plc), as previously described^13^.

### Comparative gene expression profiles of GB and healthy brain

**Figure 1:**
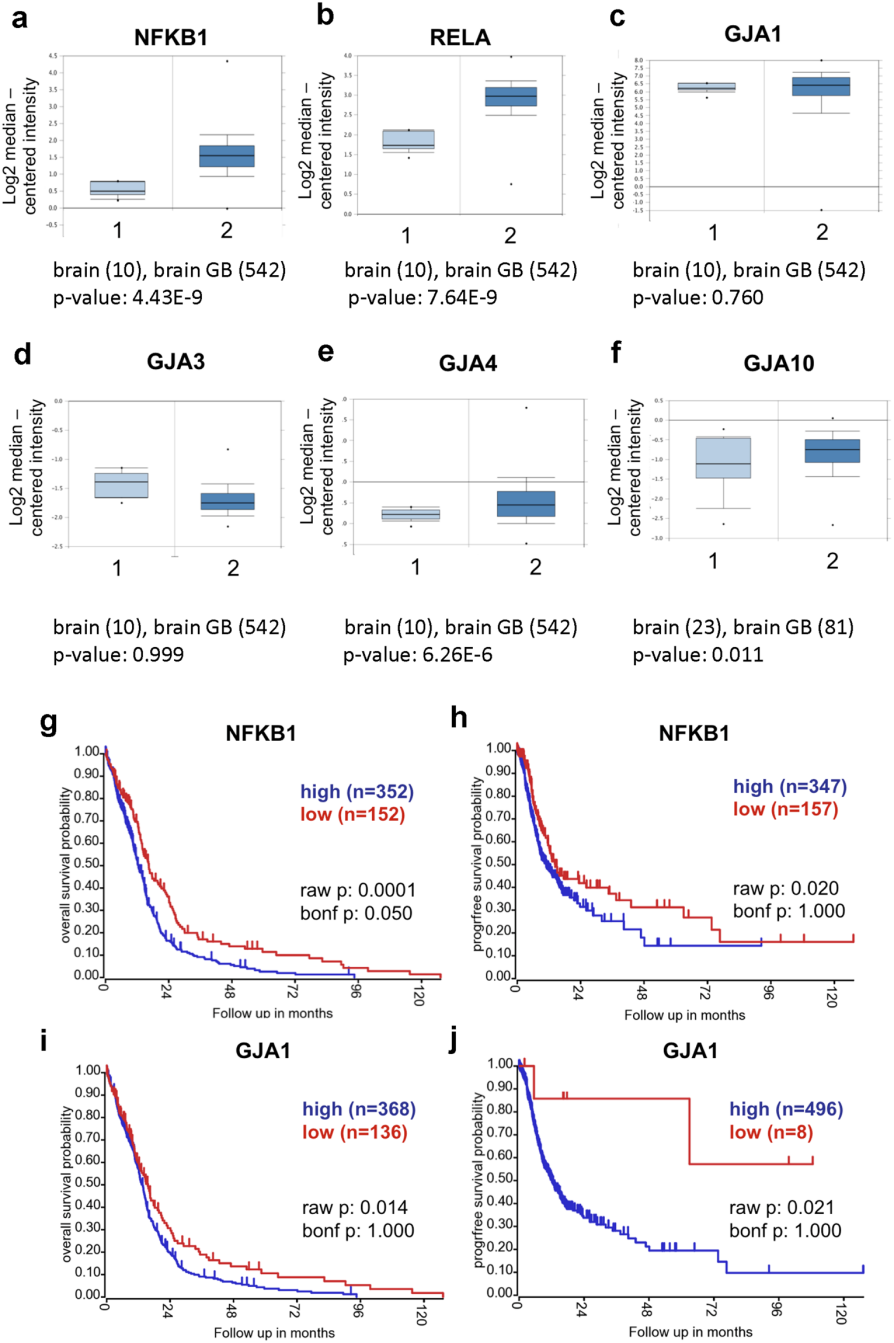
Expression of NF-κB and gap junction genes in GB and their impact on overall patient survival. Comparative gene expression [brain (1) vs. GB (2)] of NF-κB family member genes NFKB1 (p < 0.001) (**a**), RELA (p < 0.001) (**b**), Gap junction genes GJA1 (not significant) (**c**), GJA3 (not significant) (**d**), GJA4 (p < 0.001) (**e**) and GJA10 (p < 0.05) (**f**) was visualized using Oncomine Software. Sample size: as indicated. High expression of NFKB1 (**g**) and GJA1 (**i**) is a negative prognostic factor for the overall survival in GB patients. GB patients with NFKB1 (**h**) and GJA1 (**j**) high expression also negatively affect disease-free survival. Kaplan-Meier curves were prepared by the R2 software in the scan cut-off mode. In addition to the raw p-values (raw p), the adjusted p-values after Bonferroni correction are given (bonf p), which require a stricter significance threshold.

Gene expression data were analysed via the Oncomine Software (www.oncomine.com, May 2016, Thermo Fisher Scientific, Ann Arbor, MI), which is available to the public^23^. Reporter details: Fig. 1a – NFKB1 - Reporter 209239_at (TCGA Brain), Fig. 1b – Rela - Reporter 201783_s_at (TCGA Brain), Fig. 1c – GJA1 - Reporter 201667_at (TCGA Brain), Fig. 1d – GJA3 - Reporter 208590_x_at (TCGA Brain), Fig. 1e – GJA4 - Reporter 40687_at (TCGA Brain) and Fig. 1f – GJA10 - Reporter 1553044_at (Sun Brain).

### GB patient survival data

Overall and progression free survival data were obtained from the R2: Genomics Analysis and Visualization Platform (r2.amc.nl), which is available to the public. The raw data was retrieved from the GB TCGA data set (TCGA–540–MAS5.0–u133a; R2 internal identifier: ps_avgpres_broadGB540_u133a) and used for the analysis within R2. The expression cut-off for NFKB1 (209239_at) was 50.7, and for GJA1 (201667_at) 160.1 (event free survival and 1219.3 (overall survival), subset: WITH_SURV.

### Statistical analysis

Unless otherwise stated, statistical significance was assessed by either one-way ANOVA or two-way ANOVA followed by Bonferroni’s post-test to compare combination treatment with single treatments and control using the GraphPad Prism software. The Fisher’s Exact test was used in the Oncomine Software analysis to determine statistical significance. The p-values determined by the R2 software were obtained by log-rank tests.

Statistical significances are depicted as *p < 0.05, **p < 0.01 and ***p < 0.001.

### Ethical approval and consent to participate

Obtaining tumour specimens for culturing GB stem cell-like cells was approved by the Ethics Committee, Medical Faculty, Ulm University. All animal experiments were approved by the Regierungspräsidium Tübingen, Germany. All methods were performed in accordance with the relevant guidelines and regulations.

## Results

### Clinical relevance of targeting NFkB and gap junction in GB patients

Our previously published results strongly suggest that DIS and CBX affect NF-κB-mediated cell-substrate adhesions and gap junction-mediated cell-cell interactions in GB cells, respectively (for further support see Fig. S1 and Fig. S2)^13,17^. To strengthen further the validity of targeting NF-κB and gap junction in GB therapy, we hypothesized that a) GB cells should express higher levels of components of the NF-κB signalling machinery and gap junction-forming connexin proteins than healthy brain and b) there should be a correlation between NF-κB molecules and connexin on the one hand and tumour aggressiveness and reduced survival on the other. We, therefore, turned to publicly available databases (Oncomine^TM^ and R2, see Materials and Methods for details) and could verify that expression of NFKB1 (encoding the NF-κB p105 subunit), RELA (p65 subunit) and gap junction genes such as GJA4 (gap junction alpha-4 protein, Connexin 37) and GJA10 (gap junction alpha-10 protein, Connexin 62) were significantly higher in GB (n = 542) than in healthy brain (n = 10) (Fig. 1a,b,e,f). In contrast, GJA1 (gap junction alpha-1 protein, Connexin 43), GJA3 (gap junction alpha-3 protein, Connexin 46) were similarly expressed in healthy and diseased brains (Fig. 1c,d). This is to be expected even though putative roles for Connexin 43 and 46 in GB biology have previously been identified^24,25^. Connexin 43 also has a vital function in the healthy brain, such as mediating astrocyte survival^26^, while Connexin 46 so far has only been identified in GB stem TiCs which only contribute a minute fraction to the overall tumour bulk and, thus, is unlikely to appear upregulated in total GB^24^. However, both Connexin 43 and 46 are more highly expressed in GB than in neural stem cells (Fig. S3). Connexin 37 is mainly considered a vascular protein present in brain blood vessels^27^. It is tempting to speculate that its increased expression reflects increased vascularization within the tumour. Interestingly, there have been no reports linking Connexin 62 to tumour biology; it appears to be associated with connexin hemichannels of horizontal cell dendrites in the retina^28^.

Importantly, the high expression of NFKB1 and GJA1 correlated with reduced overall survival as well as with reduced progression-free survival when comparing 504 GB patient samples (Fig. 1g–j).

### Determination of DIS and CBX concentrations for combinatorial treatment of undifferentiated and stem-cell like primary GB cells

**Figure 2:**
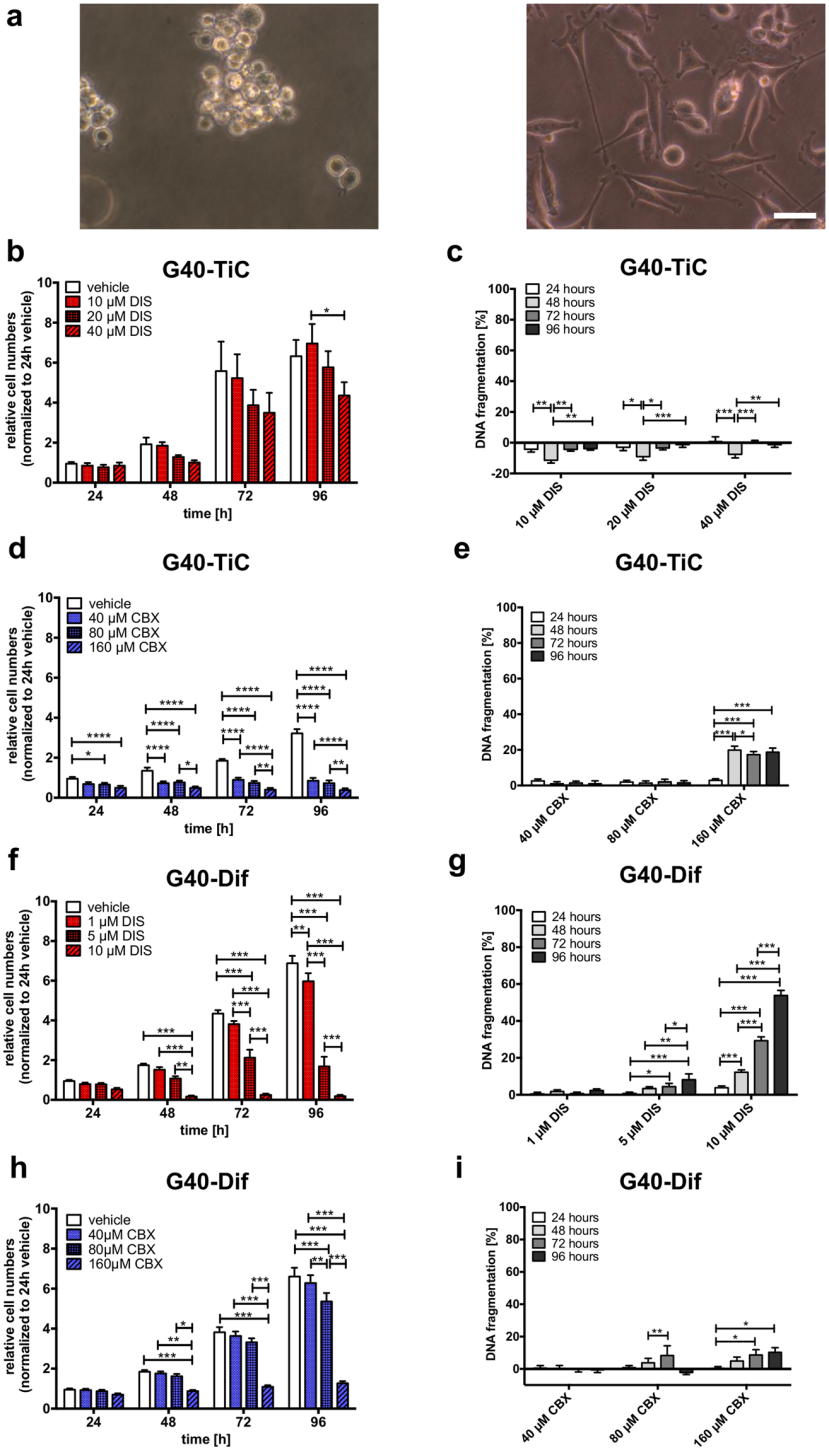
Effect of Disulfiram and Carbenoxolone on G40-TiC and G40-Dif GB cells. (**a**) Shown are primary tumour-initiating cells G40 (G40-TiC) cultured as free-floating clusters (left) and adherent differentiated GB cells (G40-Dif), which were derived from G40-TiC (right). (**b**) G40-TiC were either left untreated (exposed to DMSO solvent, vehicle) or treated with indicated concentrations of Disulfiram (DIS) and were counted every 24 h for a total of 96 h using Cell counter Casy1. Untreated controls after 24 h were defined as 1. (**c**) G40-TiC cells were cultured either in the presence or absence (vehicle, exposed to DMSO) of DIS for the indicated time points. Percentage of DNA-fragmentation of propidium iodide-stained nuclei, a surrogate for apoptosis commitment, was determined by FACS analysis. (**d**) G40-TiCs were either treated with water (vehicle) or with indicated concentrations of Carbenoxolone (CBX). The cell number was measured at indicated times whereas untreated controls after 24 h were defined as 1. (**e**) CBX-induced DNA-fragmentation was measured by FACS analysis. (**f**) G40-Dif cells were seeded and treated with vehicle (DMSO control), 1, 5 and 10 µM DIS, followed by cell counting at indicated times, where cell numbers were normalized to control vehicle at 24 h. (**g**) Percentage of specific apoptosis was determined by FACS analysis after 24 h, 48 h, 72 h and 96 h treatment with indicated concentrations of DIS. (**h**) G40-Dif cells were either left untreated (exposed to water, vehicle) or treated with 40, 80 and 160 µM CBX. Cell number was determined at indicated time points. (**i**) Specific apoptosis was measured by FACS analysis of DNA fragmentation of propidium iodide-stained nuclei after 24 h, 48 h, 72 h and 96 h. (**a**) Scale bar: 50 µm. (**b**)–(**i**) Mean and SEM of three replicates of three independent experiments are shown. Statistical significance was determined by Two-way ANOVA followed by Bonferroni’s post-test (*p < 0.05, **p < 0.01, ***p < 0.001). DNA fragmentation [%] was calculated as: 100 × (experimental apoptosis (%) - spontaneous (vehicle) apoptosis (%))/(100%-spontaneous (vehicle) apoptosis (%)).

To further elucidate the roles of cell-cell and cell-substrate interactions in GB we selected the previously characterized, patient-derived G40 cells. G40 cells are negative for MGMT protein, i.e. considered resistant to TMZ-induced apoptosis, and highly invasive in an orthotopic mouse model^21,22^. Two distinct populations were created: the spheroid-forming G40-TiC and the adherently cultured G40-Dif. While these populations represent two distinct differentiated populations, they are also representative of different dominant forms of cellular interactions: The G40-TiC mainly form cell-cell interactions, while the G40-Dif adhere to ECM-substratum (Fig. 2a).

First, we determined the optimal concentration of DIS and CBX to be used for combinatorial treatment. G40-TiC and G40-Dif cells were treated for up to 96 hours with either DIS for the inhibition of focal adhesions or the gap junction inhibitor CBX. The slight reduction in G40-TiC cell number, only significant at 96 hours after treatment with 40 µM of DIS, is unlikely to be due to cell death, as no apoptosis could be observed (Fig. 2b,c). Indeed, the negative values in specific apoptosis are indicative for a potentially protective role of DIS, possibly due to the reduction in proliferation, which also can reduce spontaneous apoptosis. In contrast CBX induced a rapid decline in G40-TiC cell numbers and a significant commitment to apoptosis after 48 hours (Fig. 2d,e). G40-Dif cells responded to DIS treatment even at a concentration of 1 µM which was detected by a decrease in cell number and apoptosis commitment at a concentration of 10 µM as early as 48 hours post treatment (Fig. 2f,g). 1 µM DIS was sufficient to prevent the translocation of p65 from the cytosol to the nucleus even after stimulation with TNF-α, hence leading to an inhibition of NF-κB signalling (Fig. S1a). While the effect of CBX on G40-Dif cell numbers appears strong, less apoptosis is induced than in G40-TiC cells (Fig. 2h,i respectively). A concentration of 160 µM CBX was sufficient to inhibit gap junctions irreversibly (Fig. S1b). This concentration is well within the range of what is used experimentally as single dose treatment and which might be achieved in the patient by multiple smaller doses^29,30^. In contrast, Chekeni and co-workers, for example, used 500 µM CBX^31^.

Taken together these data show that suspension-grown G40-TiC cells are much more resistant to the effects of DIS than adherent G40-Dif cells, while both cells show sensitivity towards concentrations of CBX within the commonly used range.

### Effect of combinatorial treatment of DIS and CBX on undifferentiated and stem-cell like primary GB cells on cell numbers, apoptosis and cell morphology

**Figure 3:**
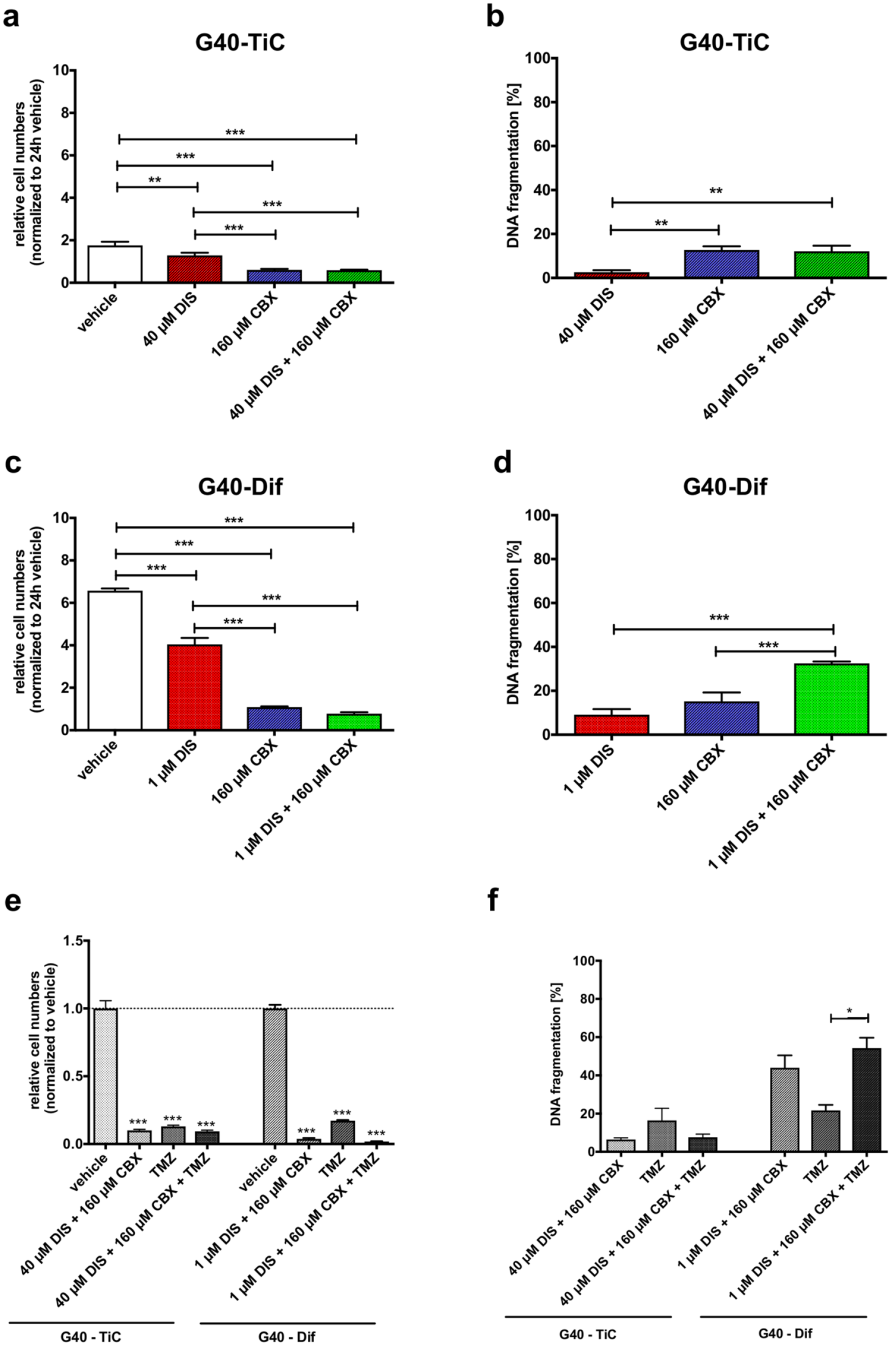
Combinatorial treatment of DIS and CBX reduces cell growth and induces apoptosis of GB cells. (**a**) G40-TiCs were seeded and immediately treated with indicated concentrations of DIS and CBX for 96 hours. A change in cell numbers was determined by the use of Casy1 cell counter, whereas the cell number of untreated cells (DMSO and H_2_O_bid_, vehicle) after 24 h was defined as 1. (**b**) G40-TiCs were treated for 96 h with DIS, CBX or both as indicated. Staining for propidium iodide was performed prior to flow cytometric analysis. (**c**) G40-Dif cells were cultured either in the presence or absence of DIS and CBX for 96 h, before cell number was measured. Untreated controls after 24 h were defined as 1. (**d**) G40-Dif cells were seeded and subsequently treated with indicated concentrations of DIS and CBX or control vehicle (DMSO and H_2_O_bid_). Percentage of DNA-fragmentation of propidium iodide-stained nuclei was visualized via flow cytometry. (**e**) GB cells, either G40-TiC or G40-Dif were either treated with vehicle, the combination of DIS and CBX used in (a)-(d), 100 µM TMZ or a combination of all three substances. A change in cell numbers was determined after 144 h by the use of Casy1 cell counter. (**f**) GB cells, G40-TiC or G40-Dif were either treated with vehicle, the combination of DIS and CBX used in (a)-(d), 100 µM TMZ or a combination of all three substances. Percentage of DNA-fragmentation of propidium iodide-stained nuclei was visualized after 144 h via flow cytometry. Mean and SEM of three independently conducted experiments in triplicate are depicted. Statistical significance was determined by One-way ANOVA followed by Bonferroni’s multiple comparison test (*p < 0.05, **p < 0.01, ***p < 0.001, ns: not significant (p > 0.05)). DNA fragmentation [%] was calculated as: 100× (experimental apoptosis (%) - spontaneous (vehicle) apoptosis (%))/(100%-spontaneous (vehicle) apoptosis (%)).

Based on experiments with the individual substances, as well as a titration analysis of CBX in combination with DIS (Fig. S4), the concentration of DIS and CBX for the combinatorial treatment was determined. The lowest concentration with a visible effect of the first effective substance, i.e. CBX for G40-TiC and DIS for G40-Dif, was combined with the second, ineffective drug of the other cell population. The purpose of this combination was to demonstrate that the combination is not antagonistic, i.e. the benefit of one substance that acts on the one subpopulation is not counteracted by the second substance. Therefore, G40-TiC and G40-Dif cells were treated with 40 µM DIS and 160 µM CBX or 1 µM DIS and 160 µM CBX, respectively. Cell numbers declined and DNA fragmentation was increased 96 hours after treatment in cells treated with either the single substance (160 µM CBX for TiCs and 1 µM DIS for Difs) or in combination. This effect was not further enhanced in G40-TiC cells when treated in combination of DIS and CBX (Fig. 3a,b). However, a slight, but significant decrease in cell numbers and increase in apoptosis commitment was detectable in G40-Dif cells (Fig. 3c,d). Similarly, combining DIS and CBX with the commonly used chemotherapeutic TMZ did not block the efficacy of either inhibitor or DNA-damaging drug (Fig. 3e,f).

### Impact of combined treatment of DIS and CBX on cell signalling molecules

Next, we investigated the effects of the two substances on cellular signalling pathways, with particular emphasis on the Raf/Mek/Erk and PI3K/Akt survival networks, which are dysregulated in various cancers. Furthermore, adherens junctions and ECM interactions are known activators of these pathways that are key mediators of adhesion-mediated apoptosis resistance (AMAR)^11,17^.

Analysis of survival factors in G40-TiC cells revealed a reduction in pAkt levels and an increase in caspase-3 cleavage, 24 hours after DIS and combined treatment of DIS and CBX, whereas CBX reduced pSrc, which – interestingly – did not occur upon combination treatment (Fig. S5a). As suggested by the data presented in Fig. 3, the combination of both substances had a larger effect on G40-Dif cells (Fig. S5). Here, individual substances had only a slight effect on the two pathways, but pAkt and pERK levels were significantly reduced upon combinatorial treatment of DIS and CBX. While low levels of cleaved caspase-3 were detectable after DIS treatment, DIS combined with CBX induced a stronger activation of caspase-3 (Fig. S5b). This is in support of the previously shown data, suggesting that the combination treatment has a much stronger apoptotic effect in G40-Dif cells (Fig. 3d).

Investigating proteins involved in cell cycle progression and ultimately proliferation, we found a reduction in cell cycle kinases, such as Cdc2 and Cdk2, in G40-TiC and G40-Dif cells treated with DIS and CBX (Fig. S5c,d). Here, the individual substances had almost equal effects in G40-TiC and combining them did not appear to enhance this effect further (Fig. S5c). In contrast, combining both substances had a strong effect on G40-Dif cells, while the individual components only exhibited a negligible causatum (Fig. S5d).

In conclusion, while the combinatorial use of DIS and CBX was originally envisaged to target different cell populations within the tumour, and therefore the requirement for those two substances was merely not to antagonize each other, we found that the combination of DIS with CBX had a major inhibitory effect on survival and cell cycle proteins involved in proliferation and apoptosis (Fig. 3 and Fig. S5).

### Effect of combinatorial treatment of DIS and CBX on G40-Dif and G40-TiC cell morphology and adhesion

A major obstacle in successfully treating GB is the highly infiltrative nature of the malignancy, which makes localized excision practically impossible^7^. Furthermore, invasive cells are frequently associated with structures that mediate increased apoptosis resistance^10^. Therefore, we next investigated the potential of the two substances in preventing the interaction between the tumour cells and their environment.

**Figure 4:**
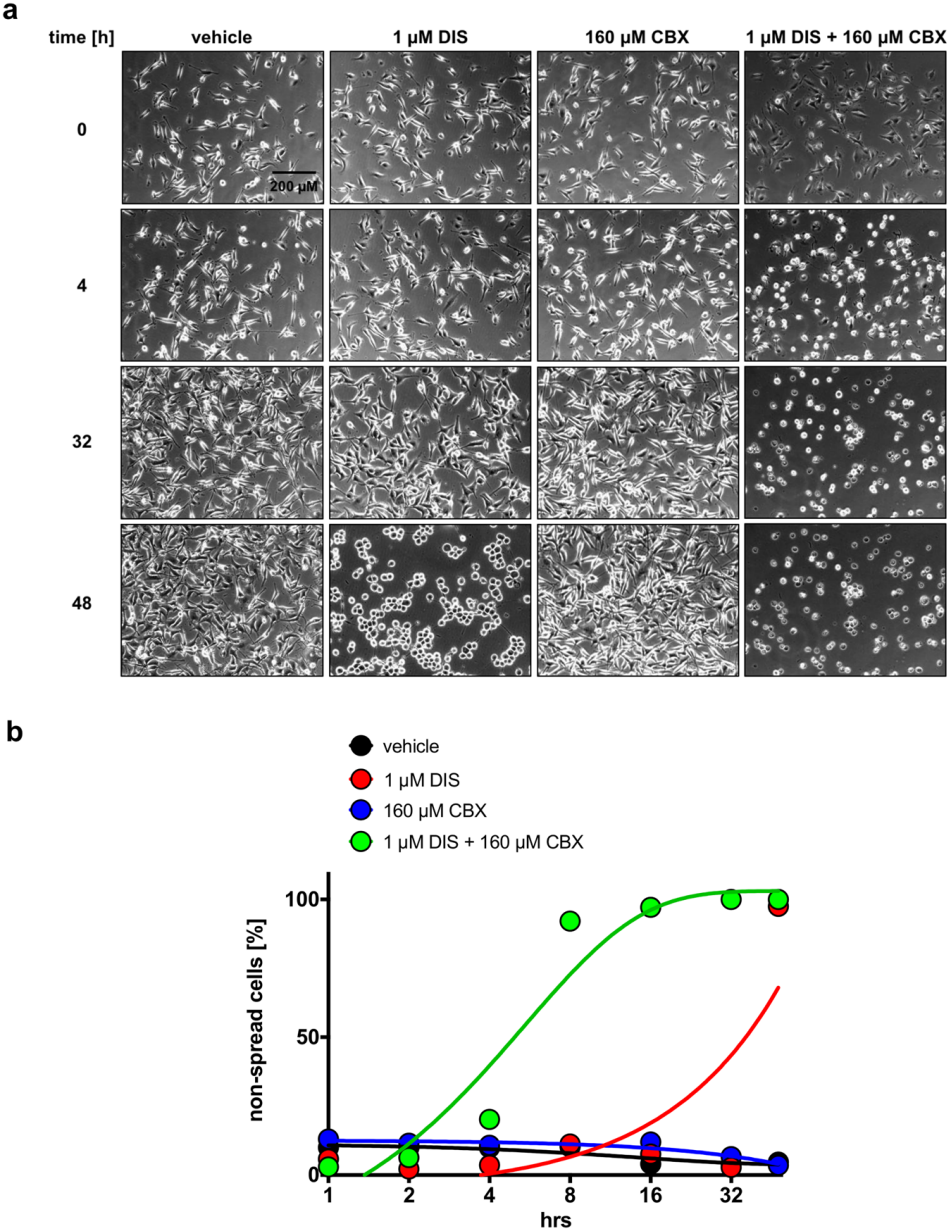
Effect of combination treatment with DIS and CBX on G40-Dif cell morphology, spreading and adhesion. G40-Dif cells were treated with indicated concentrations of DIS and CBX and pictures were taken at 10 min intervals over a time frame of 48 hours. Shown in (**a**) are representative images of two independent experiments of selected time points (0, 4, 32, 48 h). Scale bar: 200 µm. (**b**) Quantitative analysis of the cells shown in (a). The percentage of cells that were not spread at 0, 1, 2, 4, 8, 16, 32 and 48 h after treatment was calculated by counting all cells and dividing the non-spread cells by the total number of cells visible. Detailed video files have been deposited online as supplementary data.

Cell-substrate contact-forming (i.e. adherent) G40-Dif cells were examined after DIS and CBX treatment by time-lapse analysis with regards to cell morphology, spreading and adhesion. Whereas CBX treatment on its own did not have an impact on cell morphology or adhesion, 1 µM DIS was sufficient to detach the cells approximately 40 hours post treatment. By 48 hours non-adherent cell clusters have clearly formed (Fig. 4, supplementary video files). In contrast, as early as 1 hour after treatment of the cells with the combination of DIS and CBX, G40-Dif cells lost their ability to attach to the plastic and the length of the pseudofilopodia decreased in comparison to the control. Within 3–6 hours cells appeared fully detached (Fig. 4, supplementary video files). Importantly, in contrast to single treatment with DIS, the addition of CBX prevented cluster formation. Similar results were also obtained, when allowing stem cell-like cells to adhere in differentiation medium. Here 40 µM DIS prevented adhesion and thus differentiation, while the combination of DIS and CBX prevented adhesion and sphere formation (Fig. S6).

### Establishing and characterizing an adherent tumour-initiating cell model to monitor cell invasion

Next, we asked whether the different sensitivity of G40-TiC versus G40-Dif towards DIS is due to the different dominant adhesion forms, i.e. Dif cells are more sensitive as they are dependent on cell-ECM interactions, or whether the divergent differentiation status also contributes to this phenotype. To overcome this problem, we established an adherent G40-TiC cell model based on pre-existing protocols^32^, which also allowed us to investigate the locomotive potential of these stem cell-like cells. Collagen I, a compound of the ECM, is highly elevated in GB and contributes to tumour progression^33^. Hence collagen type I coated culture flasks were chosen for further investigations of the invasive phenotype of G40-TiC cells as it did not affect overall signalling of the cells (Fig. S7).

**Figure 5:**
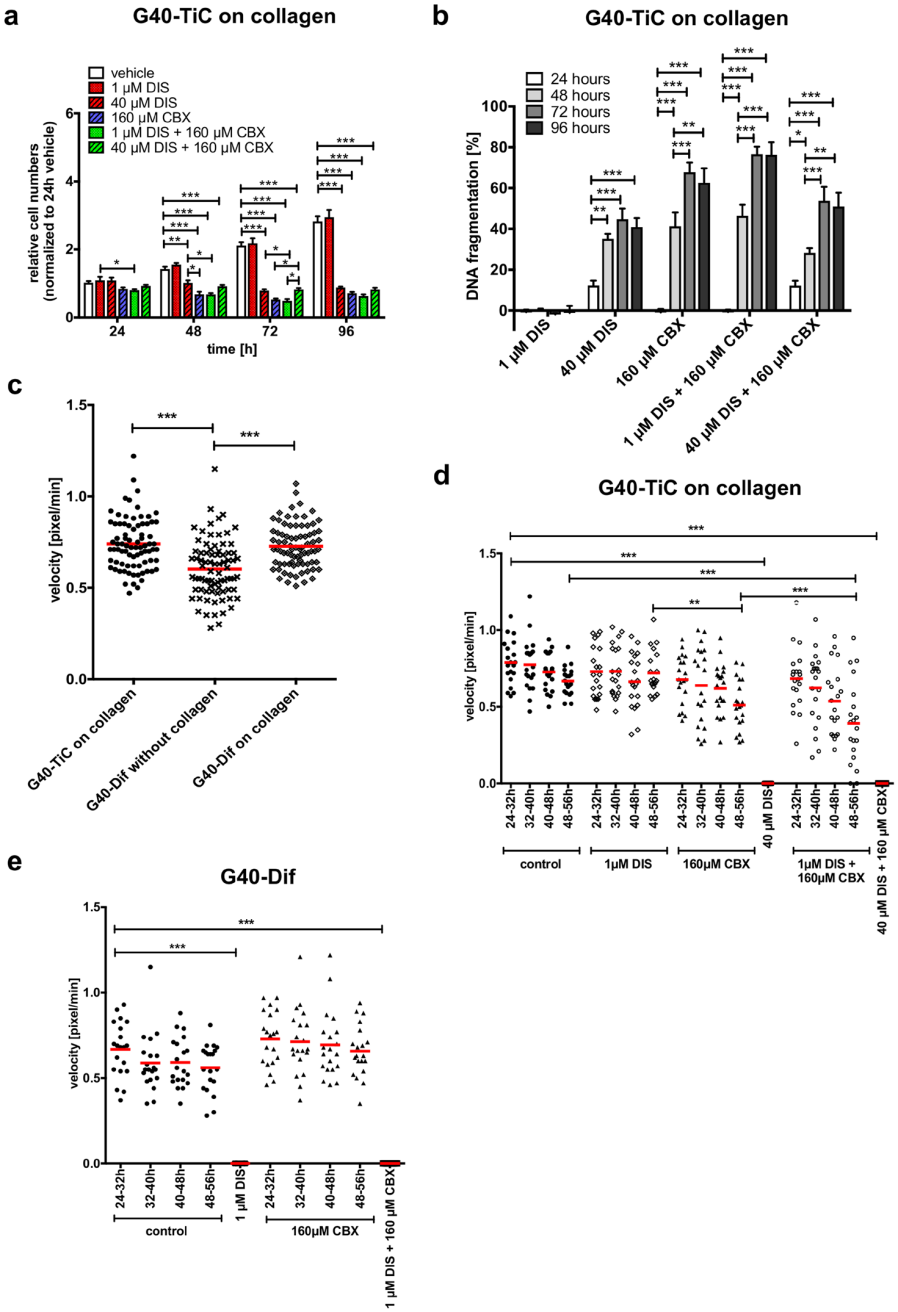
The effects of DIS and CBX on adherent G40-TiC cells and contrasting G40-TiC versus G40-Dif motility. (**a**) Effect of collagen type I on cell numbers. G40-TiCs grown on collagen were seeded and treated with DIS and CBX. A change in cell numbers was determined at the four indicated time points by the use of Casy1 cell counter. Untreated controls (vehicle) after 24 h were defined as 1. (**b**) Specific apoptosis in DIS- and CBX-treated G40-TiCs grown on collagen. G40-TiCs cultured on collagen type I were seeded and immediately treated with 1 µM DIS, 40 µM DIS, 160 µM CBX or a combination of both. This was followed by FACS analysis of the DNA fragmentation of propidium iodide-stained nuclei. (**c**) Cell velocity of G40-Dif cells cultured in the presence or absence of collagen and G40-TiCs grown on collagen was recorded every 20 minutes for 60 h via time-lapse microscopy. Migratory activity was analysed with ImageJ software (n = 80). (**d**) Cell movement of G40-TiCs cultured on collagen was examined in four 8-hour intervals (24–56 hours after seeding and immediate treatment with DIS and CBX) and analysed with ImageJ software (n = 20). (**e**) Cell velocity of G40-Dif cells was examined in four 8-hour intervals (24–56 h after seeding and immediate treatment with DIS and CBX) and analysed with ImageJ software (n = 20). Mean and SEM are depicted of three independently conducted experiments in triplicate. Statistical significance was determined by ANOVA followed by Bonferroni’s post-test (*p < 0.05, **p < 0.01, ***p < 0.001, ns: not significant (p > 0.05)). DNA fragmentation [%] was calculated as: 100× (experimental apoptosis (%) - spontaneous (vehicle) apoptosis (%))/(100%-spontaneous (vehicle) apoptosis (%)).

Adherent G40-TiC cells did not display increased sensitivity towards low concentrations of DIS (1 µM), suggesting that DIS sensitivity is not a mere consequence of adhesion (Fig. 5a,b,d). Interestingly, these cells did become more sensitive towards high concentrations of DIS (40 µM), CBX and the combination of both drugs (Fig. 5a,b; for complete statistical analysis see Supplementary Table S1).

Next, cell motility of adherent G40-TiC cells, G40-Difs and G40-TiCs was monitored via time-lapse microscopy. G40-TiCs on collagen type I coated culture flasks migrated significantly faster than G40-Dif cells, although when cultured on collagen, G40-Dif cell velocity was similar to that of G40-TiC cells on collagen, indicating that collagen had an impact on the motile phenotype (Fig. 5c). Furthermore, cell velocity upon DIS, CBX and combined treatment was analysed. None or only slight effects on cell velocity were detectable in 1 µM DIS or CBX treated G40-TiC cells (Fig. 5d). However, 1 µM DIS and 160 µM CBX combined slowed down cell movement significantly after 48 hours, whereas no migration of G40-TiC cells on collagen was detected when treated with 40 µM DIS alone or in combination with CBX. Importantly, similar to CBX, high concentrations of DIS and the combination also affect the apoptosis commitment of the adherent G40-TiCs (Fig. 5b), the possibility cannot be fully excluded that it is not the machinery of motility that is directly affected, but the general viability of the cells. However, there is no clear correlation between increase in apoptosis commitment and decrease in velocity (compare Fig. 5b with d).

On the other hand, CBX treatment on its own had no impact on G40-Dif cell migration, whereas 1 µM DIS and the combination of DIS and CBX was sufficient to detach cells at 24 hours and therefore ablated the invasive phenotype completely (Fig. 5e). Notably, the effect of DIS and CBX in G40-Dif cells was independent of the culture conditions (Fig. S8).

Taken together these data indicate that, while stem cell-like GB cells are more resistant to the effects of DIS, combining this substance with CBX, particularly at high concentrations, sensitizes these cells for cell death and reduces total numbers of tumour cells. Importantly, DIS and CBX combined have an anti-invasive effect on both cell populations investigated.

### Validating the DIS and CBX combination approach

**Figure 6:**
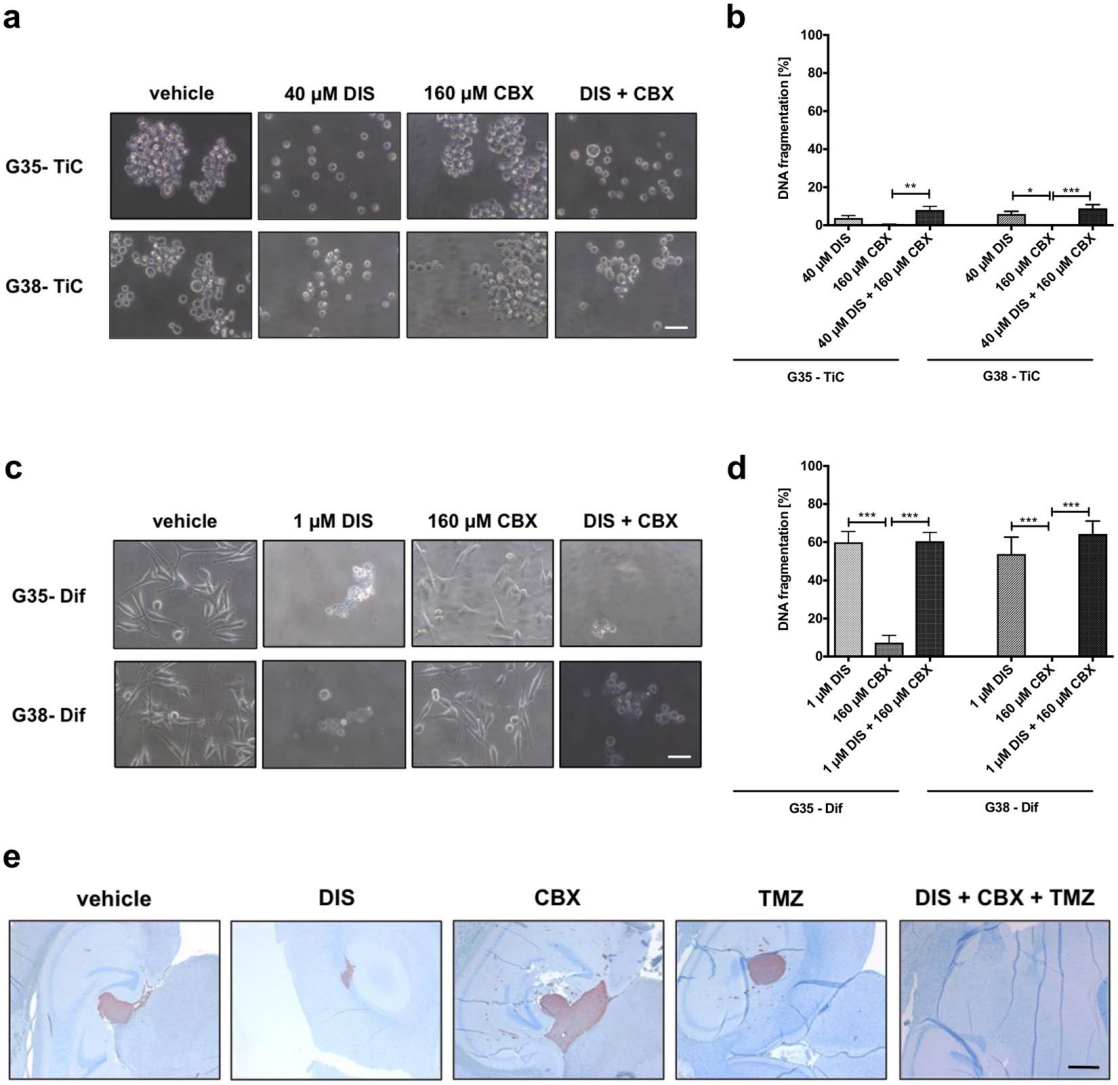
The effects of DIS and CBX on additional GB cell populations and their therapeutic potential evaluated *in vivo* TiCs (**a**,**b**) or Difs (**c**,**d**) derived from two additional patients, G35 and G38, were treated with concentrations of DIS, CBX or a combination thereof, similarly to those used with G40 cells for 96 h. (**b**–**d**) Staining for propidium iodide was performed prior to flow cytometric analysis. Mean and SEM are depicted of three independently conducted experiments in triplicate. Statistical significance was determined by One-way ANOVA followed by Bonferroni’s post-test (*p < 0.05, **p < 0.01, ***p < 0.001, ns: not significant (p > 0.05)). DNA fragmentation [%] was calculated as: 100× (experimental apoptosis (%) - spontaneous (vehicle) apoptosis (%))/(100%-spontaneous (vehicle) apoptosis (%)). (**e**) Mouse brain sections of similar depth, showing the maximal tumour bulk. Twelve mice (at least two per treatment group) were injected orthotopically with human G35-TiC cells (visualized red by Vimentin), left untreated or 7 days post injection treated with indicated substances or combinations thereof. Mice were sacrificed 11 days post injection. Concentrations were selected according to previously described literature: DIS^13^, CBX/ INI-0602^29^ and TMZ^22^. Importantly, the chosen TMZ concentration alone previously did not prevent GB-associated death in an identical experimental system^22^. (**a**),(**c**) scale bar: 50 µm. (**e**) scale bar: 1000 µm.

To verify that the observed effects of DIS and CBX are not specific for G40 cells, but represent a more general feature of GB cells, we investigated the effects of these substances on two additional pairs of highly invasive, TMZ-resistant patient-derived cell populations, G35 and G38^21,22^ (Fig. 6a–d). As with the G40 cells we observed that G35-TiC and G38-TiCs are less sensitive towards DIS-induced apoptosis than G35 and G38-Dif cells (Fig. 6b–d). However, both additional TiC populations reacted less strongly towards CBX-induced apoptosis than G40-TiCs, CBX still reduced total cell numbers of G35-TiC and G38-TiC by approximately 30% (Fig. S9). Importantly, combining both substances also did not reduce the effect of the individual substances, thus confirming no adverse drug-drug interactions upon anti-tumour efficacy.

Next, we investigated the effects of DIS and CBX in an *in vivo* setting. G40 cells are highly lethal in an orthotopic mouse model as they do not form a solid tumour^13,21^. We therefore selected the G35-TiC cells and implanted them into mouse brains. 7 days after implantation, mice were treated interperitoneally with DIS, INI-0602 – a variant of CBX optimized to cross the blood-brain barrier, but with otherwise similar chemical properties^29^, TMZ or a combination of all three substances. After sacrificing the animals 11 days post operation (4 days post treatment) tumour formation was analysed (Fig. 6e). As expected from previous data, DIS had a strong antitumor effect on the GB tumour bulk^13^, while surprisingly tumours treated with CBX appeared larger. It is tempting to speculate that this is due to the cells being less densely packed within the bulk, as treatment with CBX alone had no discernible effect on overall survival (Fig. S10). Importantly, no tumour bulk was detectable upon triple treatment with DIS, CBX and TMZ (Fig. 6e).

From previous experience (data not shown), it is to be deduced that when treatment was given at Day 7, GB cells were already invasive. In addition, untreated mice already began dying at Day 17 (Fig. S10). Accordingly, invasive cells were found in all brain sections analysed independently of treatment. However, the morphology of invasive cells exposed to triple treatment was noticeably different, and cells appeared considerably smaller and rounded, indicating reduced locomotive capacity and/or viability.

Taken together, these data strongly suggest that the addition of DIS and CBX to the standard therapeutic approach for GB treatment might have a beneficial effect, affecting both the size of the tumour bulk and the invasive potential of individual cells.

## Discussion

Due to its unfocused growth and highly invasive nature, therapeutic options for GB are severely limited. Localized treatment, such as surgical resection and radiotherapy, might successfully eliminate the tumour bulk, but are unlikely to affect the tumour cells dispersed throughout the brain, leading to rapid recurrence^4^. The application of systemic chemotherapy, however, is highly limited due to the blood-brain-barrier. The only chemotherapeutic routinely given to GB patients, TMZ, only prolongs patient’s life expectancy by a few months^34^. This sobering fact might be due to several reasons, not least the association of invading GB cells with brain structures that increase apoptosis resistance^9,10^. Clearly, new conceptual approaches to GB therapy are needed.

Taking our cue from the famous prison island of Alcatraz, which put several (overlapping) isolating circles between the prisoners and society, we postulate the Alcatraz Strategy: Isolating GB from their direct microenvironment within the brain, and, thus, reducing invasion and turning GB into a more focused disease that is less therapy resistant.

Here, we selected two of those interactions, cell-substrate adhesion and gap junctions and using genetically identical cell populations, which – due to their alternative differentiation statuses – expressed different forms of dominant cellular interactions to test our hypothesis. Blocking gap junctions, which are found in suspension-cultured tumour-initiating cells (TiCs) and – although of a more transient nature – in differentiated cells (Difs), affected apoptosis and total cell numbers in both cell populations. In contrast, blocking cell-substrate interaction only affected the Dif population. Even concentrations of the inhibitor Disulfiram (DIS) far exceeded the physiologically relevant doses, had only minor effects on the TiC cell clusters. The latter is to be expected, as under the given culture conditions these cells do not adhere to substrate. However, under induced adherence (which did not lead to differentiation) these cells remained resistant to DIS treatment – suggesting that adhesion in TiCs is somehow fundamentally different from adhesion in Difs. This is in line with our recent data, suggesting that motility, i.e. dynamic adhesion turnover, is differently regulated in those two cell populations and opens interesting avenues for further research. Investigating how TiCs migrate and cataloguing potential inhibitors will enhance our general knowledge of GB biology and identify additional components of the Alcatraz Strategy.

Importantly, combining the cell-substrate inhibitor DIS and the gap junction blocker Carbenoxolone (CBX), either with only each other or additionally with TMZ, does not inhibit the efficacy of the individual substances, i.e. they are suitable candidates for a complex combination therapy that adds to the current standard treatment.

Possibly the most striking feature of our investigation is the emergence of cooperation between DIS and CBX, where therapeutically beneficial alterations occur which are not the mere sum of the effects exerted by the individual compounds, and when blocking Dif motility. While DIS alone can effectively lead to loss of adhesion, this only occurs 40 hours after treatment initiation. As clearly visible in the time-lapse data provided online, prior to detachment, cells undergo a morphological change – increasing the surface area in contact with neighbouring cells. We speculated that this is an attempt to change the dominant form of adhesion from cell-substrate to cell-cell interaction, a phenomenon we described earlier^17^. The addition of CBX further altered the kinetics, leading to a detachment within 3 hours. Here we clearly see that the two substances we selected to affect different cell populations also strongly synergize.

An independent line of reasoning supports our notion that NF-κB and gap junctions, the direct targets of the inhibitors, are crucial for GB, which was established by mining publicly available data sets. Here, we could show that our molecules of interest are more highly expressed in GB compared to healthy brain and expression is associated with poorer diagnosis in patients, further validating our target selection.

Blocking cellular interactions as a therapeutic avenue is often viewed with scepticism for fears that this might facilitate epithelial-to-mesenchymal transition (EMT), creating a more motile phenotype of cancer cells and thus actually enhancing invasion and metastasis. Although GB cells do not undergo classical EMT, no basement membrane is present and E-cadherin is only rarely expressed in GB^35^. The gene expression profile of human mammary epithelial cells that have undergone EMT is similar to that of GB cells^36^, i.e. GB cells are already highly motile and targeting interaction points with the microenvironment is unlikely to enhance this effect further. Nevertheless, we have selected cellular interactions which are not considered to be adhesions *per se*. Furthermore, for cell-substrate interactions we have not selected a specific inhibitor, such as the integrin inhibitor Cilengitide which has not lived up to its initial therapeutic promise^12^. Instead we selected DIS, an off-patent drug that has been used in the treatment of chronic alcoholism by inhibition of acetaldehyde dehydrogenase (ALDH). It is known to be well tolerated with low toxicity and only minor side effects^37^. DIS has been shown to exert therapeutic benefits on GB cells via different mechanisms, such as blocking NF-κB, proteasome inhibition and a reduction of DNA repair pathways^13,38–40^. In turn, we utilized the DIS-inhibition of NF-κB to block cell-substrate contacts and, thus, invasion^13^, which also has additional therapeutic benefits, such as antiproliferative effects^40,41^. This could explain why, in our mouse model, we see a reduction in tumour bulk as well as reduced invasion. Importantly, this putative reduction in proliferation does not interfere with TMZ-induced cell death. This is in line with other data showing that sensitivity towards this chemotherapeutic treatment does not correlate with proliferation rates^42^. DIS is already being clinically evaluated as one of nine repurposed drugs (CUSP9) in the novel treatment approach against GB^43^, while a phase I trial recently revealed that DIS can be safely combined with TMZ in the treatment of GB patients^44^.

CBX, which is structurally related to the chief constituent of liquorice roots, is being used to treat gastric ulcers and other types of inflammation^45^. While we used CBX as a water-soluble inhibitor of gap junctions, it is relatively nonspecific also blocking other potential routes of cellular signalling, such as NMDA receptors, GABA_A_ receptors, and calcium channels^45^. Importantly, it is not a general inhibitor of connexins. While being the main proteins that make up gap junctions, connexins also have intracellular signalling functions that have been described as tumour-suppressive, e.g. acting as an inhibitor of cell growth^46^. Therefore, targeting the structure of gap junctions is preferential to targeting connexins.

CBX has also been reported to reduce seizures, act in a neuroprotective manner and improve cognitive functions^45,47,48^. These effects, not all related to its function as gap junction blocker, might have a beneficial effect on the overall well-being of GB patients.

## Conclusion

Taken together, DIS and CBX are an ideal addition to a complex combination therapy that aims to isolate GB from its direct environment. These substances have a long history of clinical use and the side effects are therefore well-understood, the most severe being alcohol intolerance (due to DIS) and hypertension (due to CBX). The latter can be countered by co-administration of amiloride^48^. However, further additions to the Alcatraz Strategy still need to be considered, as tumour cells are well-known to be able to alternate between the different forms of interactions upon stress or microenvironmental changes^11^. Crucially, this is also reflected by different forms of motility these cells can adopt, particularly under selective pressure^49,50^. Monitoring the long-term effect of DIS and CBX on GB cells is therefore necessary to identify potential alterations in the underlying molecular mechanisms of invasion that arise due to selective pressure, and warrant the augmentation of further contact-inhibiting drugs. Nevertheless, it seems clear that adding DIS and CBX to the standard treatment for GB has the potential to improve the therapeutic window, both temporally and spatially, suggesting that further clinical evaluation should commence as soon as possible.

## Electronic supplementary material

Supplementary information

Supplementary video vehicle treatment

Supplementary video DIS treatment

Supplementary video CBX treatment

Supplementary video DIS + CBX treatment
