# Supplementary material for: Blocking distinct interactions between Glioblastoma cells and their tissue microenvironment: A novel multi-targeted therapeutic approach
Source: Sci Rep. 2018 Apr 3;8:5527. doi: 10.1038/s41598-018-23592-z (PMC5882900; doi:10.1038/s41598-018-23592-z)
Supplement: Supplementary file 1 — Supplementary information [file 41598_2018_23592_MOESM1_ESM.pdf]

Supplementary materials

Blocking distinct interactions between Glioblastoma cells and their tissue microenvironment. A novel multi-targeted therapeutic approach.

Melanie Mettang, Viola Meyer-Pannwitt, Georg Karpel-Massler, Shaoxia Zhou, Neil O. Carragher, Karl Josef Föhr, Bernd Baumann, Lisa Nonnenmacher, Stefanie Enzenmüller, Meike Dahlhaus, Markus D. Siegelin, Sebastien Stroh, Daniel Mertens, Pamela Fischer-Posovszky, E. Marion Schneider, Marc-Eric Halatsch, Klaus-Michael Debatin, Mike-Andrew Westhoff

Supplementary Materials:

Additional materials

Bay 11-7082 was purchased from Selleckchem (Munich, Germany), EDTA from Sigma Aldrich (Steinheim, Germany) and GAP27 peptide form ApexBio (Houston, TX, USA). Anti-phospho-Serine/Threonine was obtained from BD Biosciences (Bedford, MA, USA), while two anti-phospho-Tyrosine antibodies (PY20, PY99) were from Santa Cruz Biotechnology, Santa Cruz, CA, USA.

Fluorescence microscopy

Fluorescence microscopy was performed as previously described[13]. Cells were seeded in chamber slides and allowed to settle o/n before being treated with Disulfiram. As positive control, some cells were stimulated with TNF-α (Biochrom, Berlin, Germany), a known activator of NF-κB. Cells were then fixed in 3.7% paraformaldehyde, permeabilized using 0.5% Triton-X and stained with the primary antibody NF-κB p65 (Santa Cruz Biotechnology), which in turn was visualized using an FITC-labelled secondary antibody (Santa Cruz Biotechnology). p65 localization was then analysed by fluorescence microscopy with an AX70 microscope from Olympus, Hamburg, Germany.

Electrophysiology

Electrophysiological experiments were performed with AdhTiC-G40 and AdhDif-G40 cells cultivated for 2-3 days. Membrane currents were recorded in the whole-cell recording mode using an EPC-9 amplifier and Patchmaster software (HEKA, Lambrecht, Germany)[51]. Before recording, cells were rinsed twice with a physiological salt solution composed of 140 mM NaCl,

5 mM KCl, 1 mM $MgCl_2$, 1.5 mM $CaCl_2$, 6 mM glucose, 12 mM Hepes, pH 7.3. Patch pipettes were drawn from borosilicate glass and filled with 125 mM KF, 1 mM $MgCl_2$, 10 mM EGTA, 10 mM Hepes, pH 7.2. All experiments were performed at room temperature. The gap junction inhibitor CBX was applied by a computer-controlled perfusion system at a concentration of 40 μM or 160 μM. Starting from a resting potential of around -30 mV a hyperpolarising voltage jump of -5mV was applied for a period of 20 ms. The current amplitude at the end of the voltage jump of the resulting current transient was used as measure for the functional status of the gap junctions. Voltage jumps were repeated every 4 seconds in the absence or presence of CBX.

Western blot analysis

Specific protein expression in cells was determined by Western blot analysis as described before[21]. Briefly, equal amounts of total proteins (50 µg) were resolved on 12% SDS-PAGE gels and transferred onto a Hybond ECL nitrocellulose membrane (Amersham Biosciences, Freiburg, Germany) using a semidry blotting system. Proteins were visualized by ECL western blotting detection reagents (Amersham Biosciences), according to the manufacturer's protocol. Following antibodies were used: rabbit anti-caspase-3 (Cell Signaling, Frankfurt, Germany), rabbit anti-phospho-Akt (Ser473) (Cell Signaling), mouse anti-Akt (BD Bioscience), mouse anti-phospho-ERK1/2 (Cell Signaling), rabbit anti-ERK1/2 (Sigma-Aldrich, Darmstadt, Germany), rabbit anti-phospho-Src family (Cell Signaling) mouse anti-Src (Cell Application, San Diego, CA, USA), mouse anti-Chk1 (Santa Cruz Biotechnology, Santa Cruz, CA, USA), mouse anti-CDK2 (Santa Cruz Biotechnology), mouse anti-CDC2 (Santa Cruz Biotechnology), mouse anti-β-actin (Sigma-Aldrich) and secondary HRP-linked antibodies were purchased from Santa Cruz Biotechnology.

Additional comparative gene expression profiles of GBM and healthy brain

Reporter details: Supl. 10a- GJA1 – Reporter 201667_at (Lee Brain), Supl. 10b - GJA3 – Reporter 239572_at (Lee Brain).

Supplementary References:

51. Hamil, O. P., Marty, A., Neher, E., Sakmann, B. & Sigworh, F. J. Improved patch-clamp techniques for high-resolution current recording from cells and cell-free membrane patches. *Pflugers Arch* **391**, 85-100 (1981).

Supplementary Figures:

Supp. Fig. 1

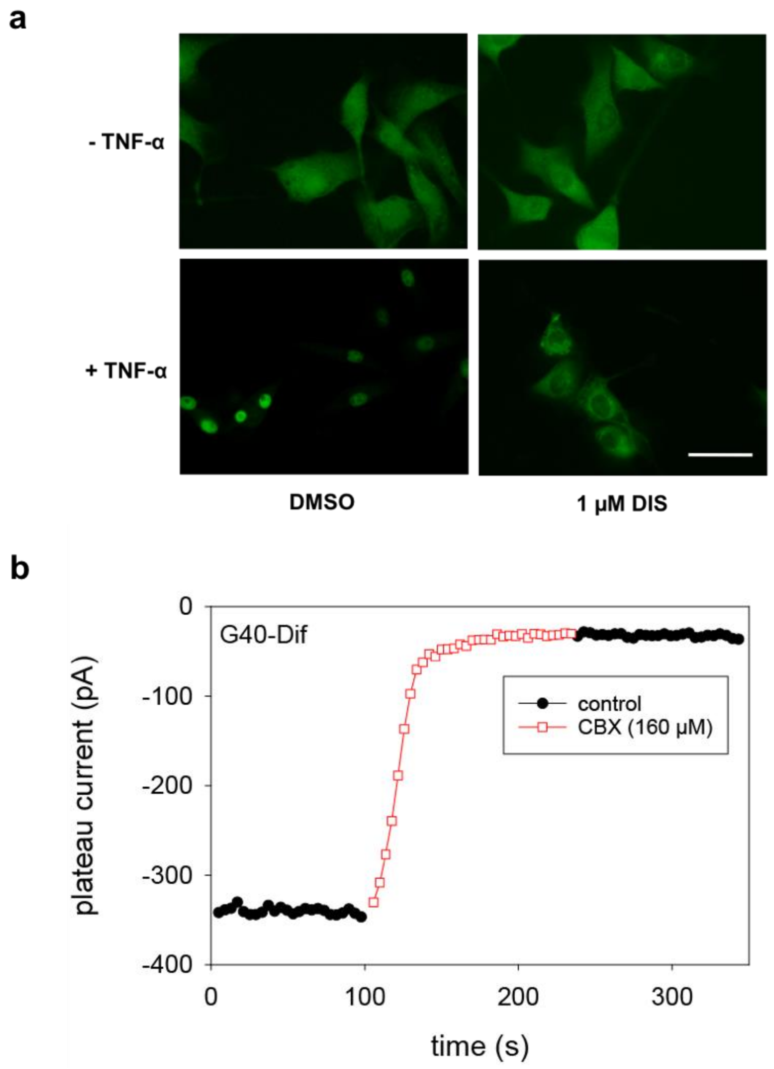

Supplementary Figure S1: DIS inhibits NF-κB translocation and CBX irreversibly closes gap junctions in GBM cells.

(**a**) G40-Dif cells were seeded in chamber slides and allowed to settle o/n. Cells were then treated with vehicle (DMSO control), 1 µM DIS for 2 hours, vehicle and 50 ng/ml TNF-α for 1 hour, or the combination of DIS and TNF-α. Cells were then fixed and stained for the NF-κB p65 subunit, whereupon p65 localization, a surrogate for NF-κB activation, was analysed by fluorescence microscopy. Scale: 50 µm. (**b**) The functional status of the gap junctions was examined by means of the patch-clamp technique. A voltage jump of -5mV was applied for a period of 20 ms, resulting in a rapidly decaying peak current and a sustained plateau current. The current amplitude at the end of the voltage jump was used as a measure for the functional status of the gap junctions. Voltage jumps were repeated every 4 seconds in the absence (black) or presence (red) of CBX. Plateau current changes in G40-Dif cells treated with 160 µM CBX. One of two representative measurements is depicted.

Supp. Fig. 2

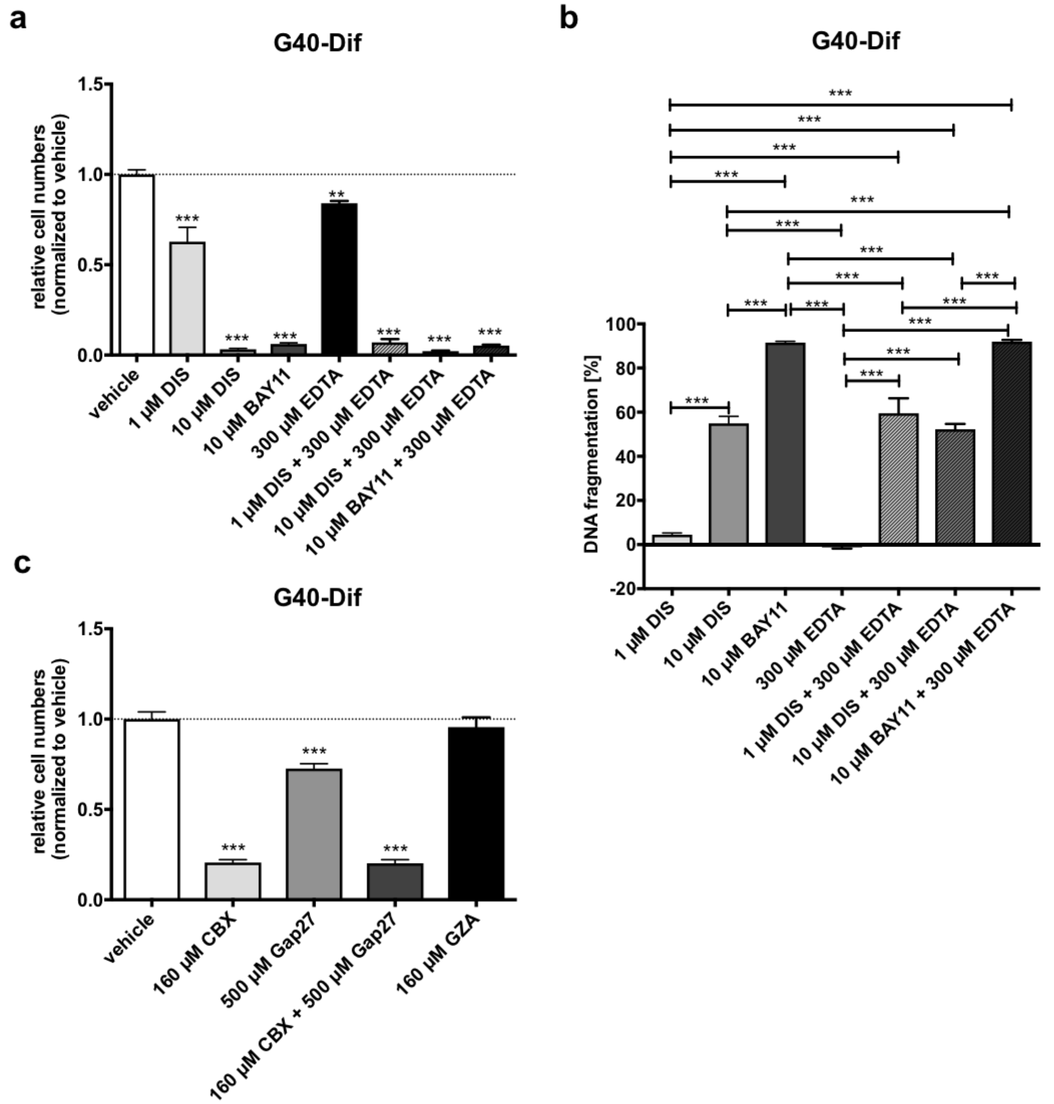

Supplementary Figure S2: Interference with cell-ECM and cell-cell interaction inhibits proliferation and induces apoptosis in differentiated GB cells.

(**a**) G40-Dif cells were seeded and immediately treated with indicated concentrations of DIS, Bay11 and EDTA. The change in cell numbers was determined 96 hours post treatment by the use of Casy1 cell counter. Bay11: Bay 11-7082, a specific NF-κB inhibitor; EDTA: a chelator of $Ca^{2+,}$ blocks calcium-dependent signalling, such as gap junctions. (**b**) Apoptosis was measured by FACS analysis of DNA fragmentation of propidium iodide-stained nuclei after 96 hours treatment similarly to (a). (**c**) G40-Dif cells were treated with indicated concentrations of CBX, Gap27 and GZA and cell numbers were determined using a Casy1 cell counter. Gap27: connexin 43 mimetic peptide that blocks channel formation; GZA: glycyrrhetinic acid (of which CBX is a derivative), which does not inhibit gap junctions.

Mean and SEM of three independent experiments performed in triplicate are shown. Statistical significance was determined by One-way ANOVA followed by either Dunnett's (*a*, *c*) or Bonferroni's multiple comparison test (*b*) (*p<0.05, **p<0.01, ***p<0.001).

Supp. Fig. 3

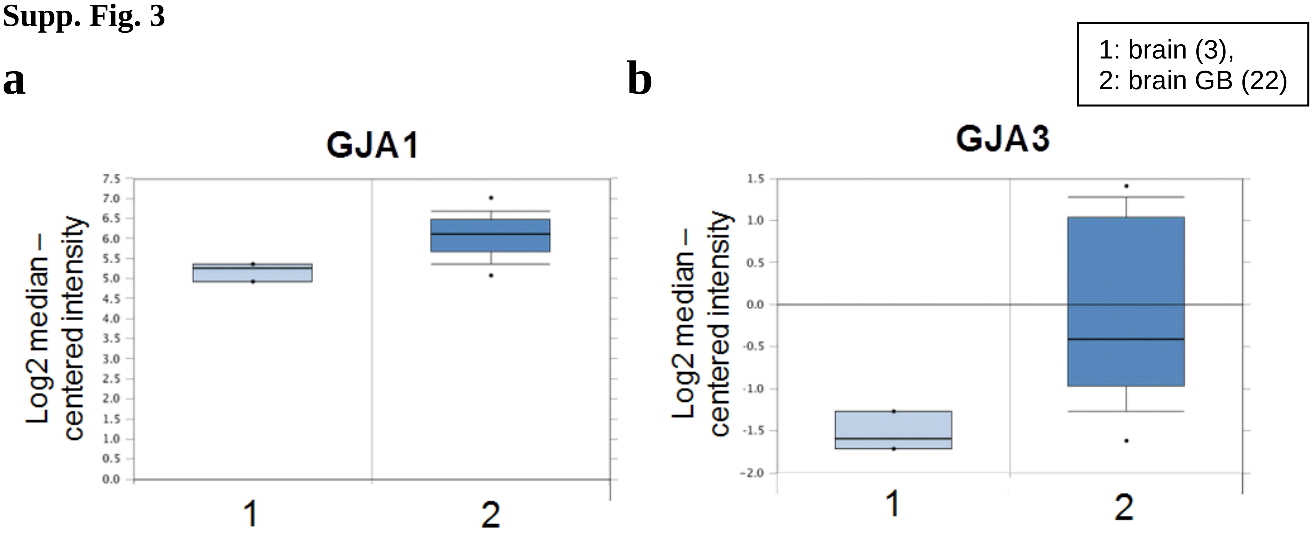

neural stem cell (3), GB (22)

p-value: 8.78E-4

neural stem cell (3), GB (22)

p-value: 2.96E-5

Supplementary Figure S3: Expression of gap junction genes in GBM and neural stem cells.

Comparative gene expression [neural stem cell (1) vs. GBM (2)] of Gap junction genes GJA1 ($p<0.001$) (**a**) and GJA3 ($p<0.001$) (**b**) was visualized using Oncomine Software. Sample size: as indicated.

Supp. Fig. 4

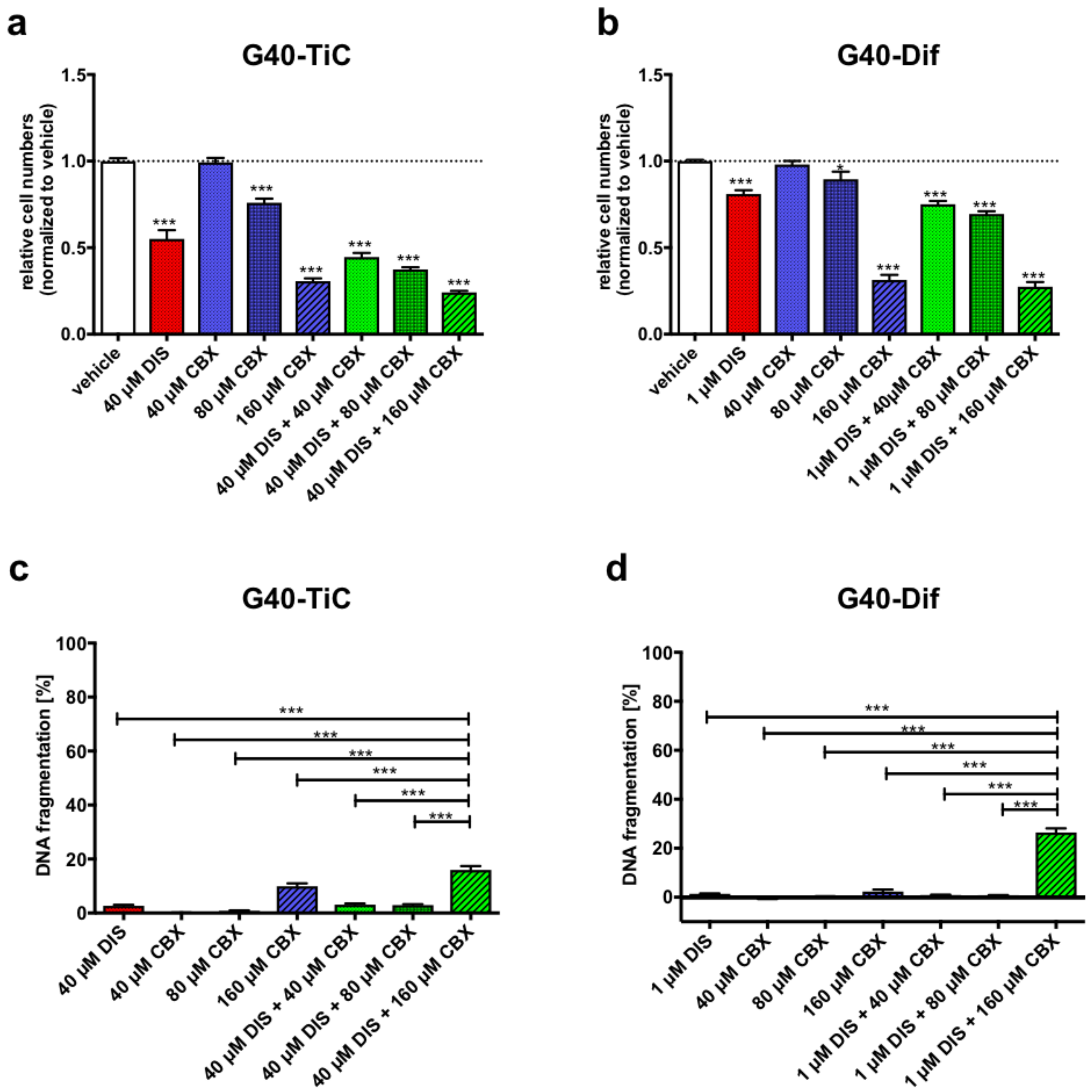

Supplementary Figure S4: Titration analysis of combination treatment with DIS and CBX on cell count and apoptosis of G40-TiC and G40-Dif cells.

G40-TiCs (**a**) and G40-Dif cells (**b**) were seeded an immediately treated with indicated concentrations of DIS and CBX for 72 hours. The cell number was determined by measuring the cell number with a Casy1 cell counter, whereas the cell number of untreated cells (DMSO and H_2O_{bid}, vehicle) was defined as 1. G40-TiCs (**c**) and G40-Dif cells (**d**) were treated with DIS and CBX as indicated. Apoptosis was measured by FACS analysis of DNA fragmentation of propidium iodide-stained nuclei after 72 hours. Mean and SEM of three independent experiments performed in triplicate are shown. Statistical significance was determined by One-way ANOVA followed by either Dunnett's (*a*, *c*) or Bonferroni's multiple comparison test (*b*, *d*) (*p<0.05, **p<0.01, ***p<0.001).

Supp. Fig. 5

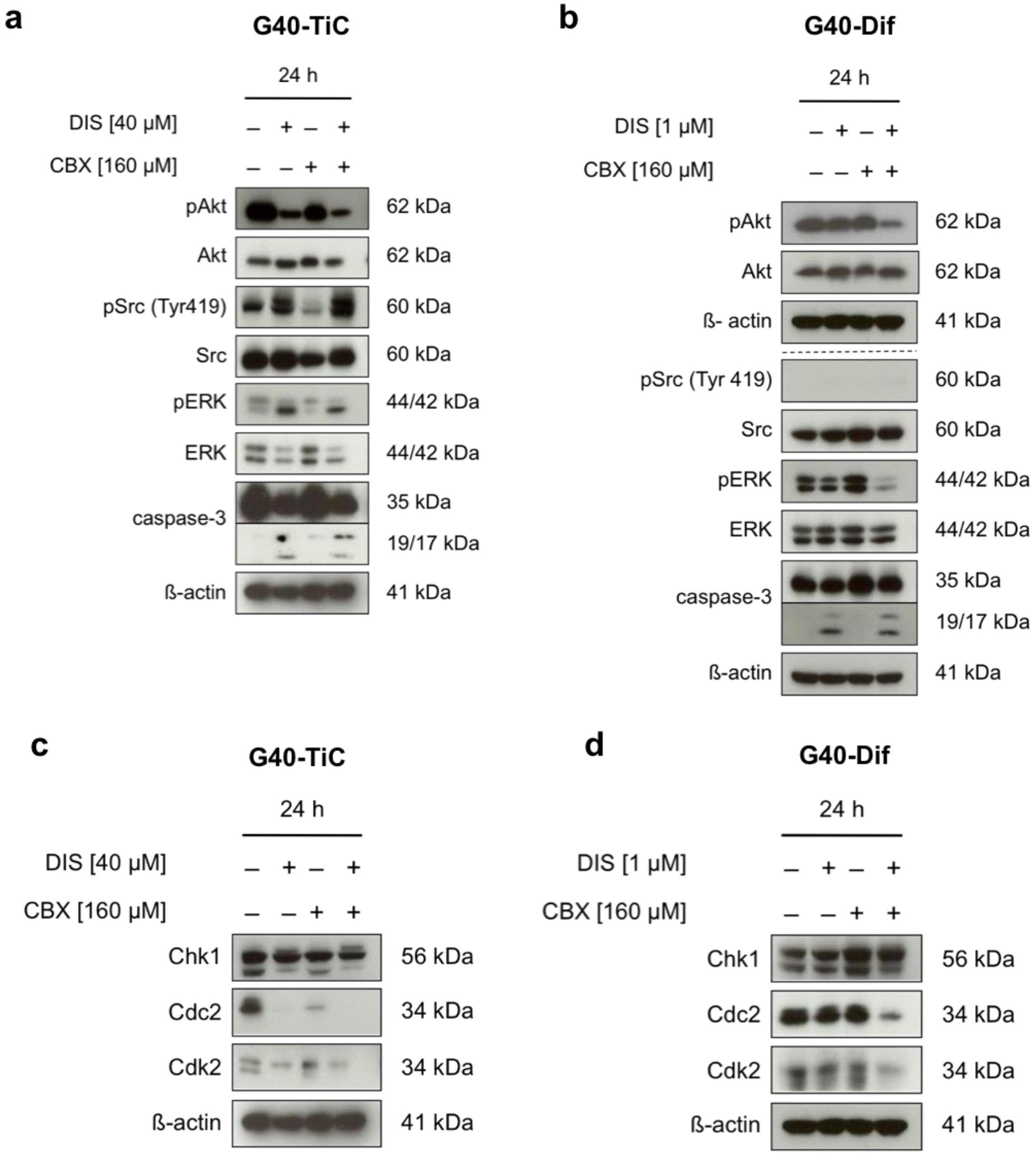

Supplementary Figure S5: Treatment of GB cells with DIS and CBX leads to downregulation of key survival and cell cycle proteins.

(**a**) G40-TiCs and (**b**) G40-Dif cells were seeded and treated for 24h with indicated concentrations of DIS, CBX and the combination of both compounds. Protein expression levels and phosphorylation status of Akt, Src, Erk and cleaved caspase 3 were analysed by Western blotting. ß-actin served as loading control. (**c**) G40-TiC and (**d**) G40-Dif GB cells were treated with DIS and CBX as indicated. Whole-cell extracts were examined by Western blot for Chk1, Cdc2 and Cdk2. ß-actin served as loading control. A representative experiment of at least two independent data sets is shown.

Supp. Fig. 6

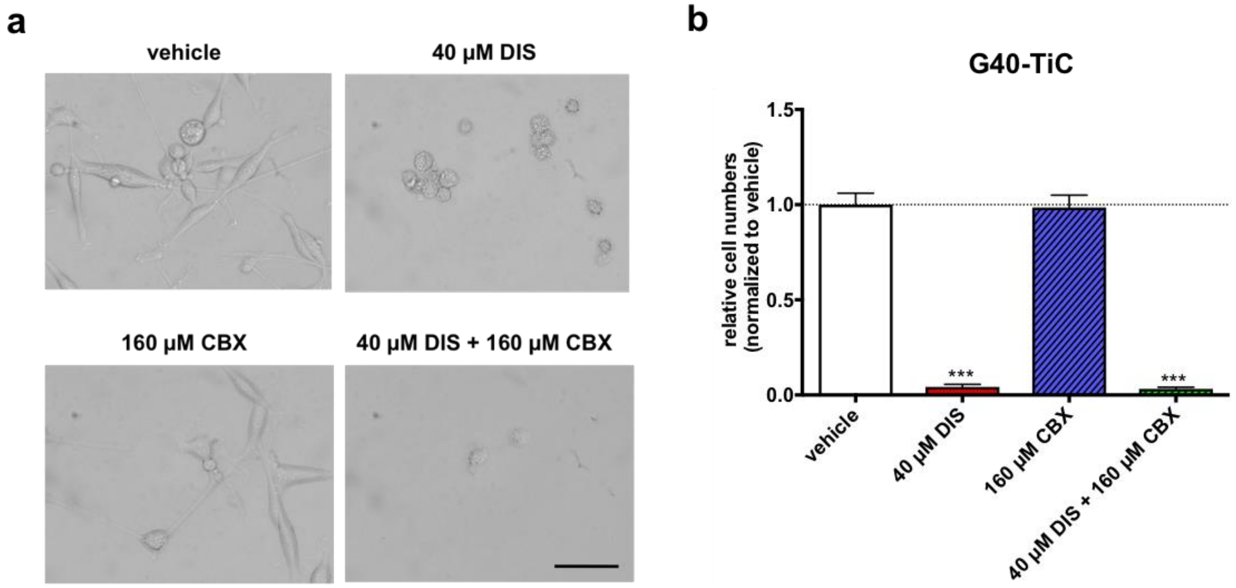

Supplementary Figure S6: Effect of combination treatment with DIS and CBX on G40-TiC morphology and differentiation.

G40-TiCs were cultured in differentiation medium and immediately stimulated with indicated concentrations of DIS and CBX. After 72 hours, pictures were taken (**a**) and cell number of the adherent cells were determined by Casy1 cell counter (**b**). Data are presented as the mean and SEM of three replicates performed in triplicate. Statistical significance was determined by One-way ANOVA followed by Dunnett's post-test (***$p<0.001$), comparing DIS and CBX stimulated cells with control vehicle (DMSO and H_2O_{bid}). (*a*): scale bar: 50µm

Supp. Fig. 7

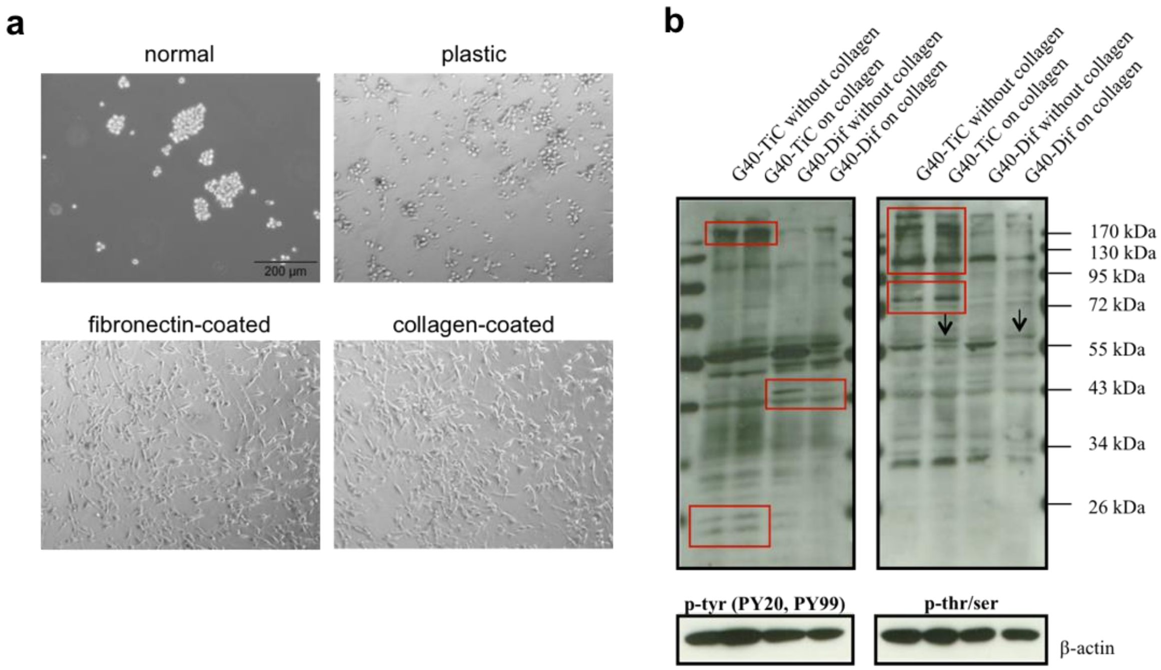

Supplementary Figure S7: Establishing of an adherent tumour-initiating cell model and analysis of phosphorylation expression patterns.

(**a**) G40-TiCs were cultured in stem cell medium in a stem cell culture flask (normal growth condition), cell culture-treated plastic (for adherent cells), a fibronectin-coated culture dish and a collagen type I-coated culture dish. Attached G40-TiC cells possess pseudopodia, but not as pronounced as G40-Dif cells. Scale: 200 µm. (**b**) G40-TiC and G40-Dif cells were cultured in the presence or absence of collagen type I and analysed for p-tyrosine and p-threonine/serine expression patterns via Western blot. Red boxes indicate similarities in phosphorylation patterns between G40-TiCs and G40-TiCs on collagen as well as G40-Dif cells and G40-Dif cells grown on collagen. Arrows indicate similarities in p-thr/ser expression patterns at about 60 kDa for different cells types grown on collagen I. Importantly, G40-TiC cultured on collagen type I did not differentiate into G40-Dif cells, but maintained their stem-cell like properties as suggested by the overall phosphorylation pattern. Depicted is one representative experiment of two.

Supp. Fig. 8

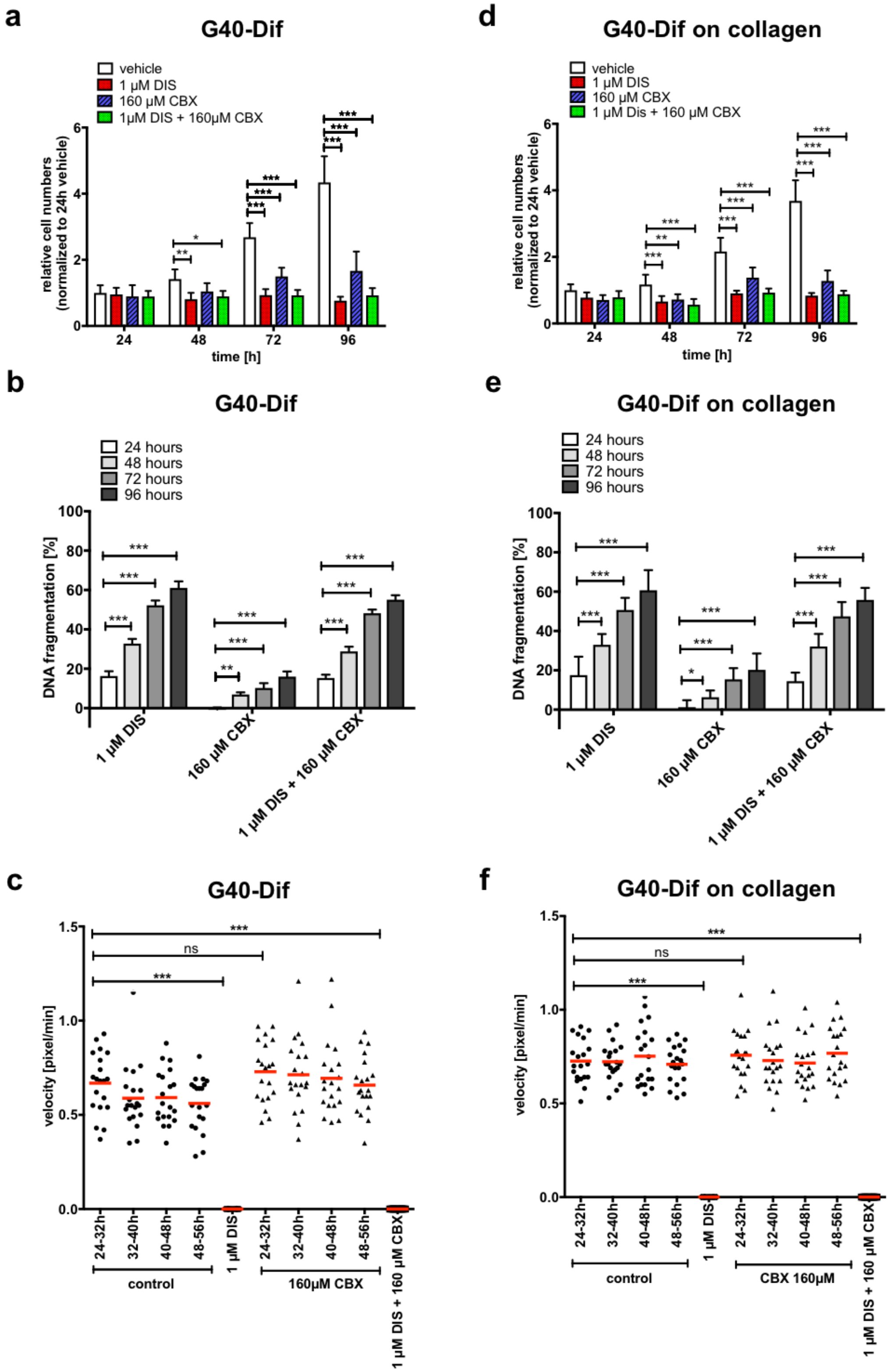
a
G40-Dif
vehicle
1 µM DIS
160 µM CBX
1µM DIS + 160µM CBX
relative cell numbers (normalized to 24h vehicle)
time [h]
d
G40-Dif on collagen
1 µM Dis + 160 µM CBX
b
G40-Dif
24 hours
48 hours
72 hours
96 hours
DNA fragmentation [%]
1 µM DIS
160 µM CBX
1 µM DIS + 160 µM CBX
e
G40-Dif on collagen
c
G40-Dif
velocity [pixel/min]
ns
24-32h
32-40h
40-48h
48-56h
control
160µM CBX
f
G40-Dif on collagen
CBX 160µM

Supplementary Figure S8: Comparing the treatment effects of DIS and CBX on cell count, apoptosis induction and cell velocity of G40-Dif cells cultured in the presence or absence of collagen type I.

G40-Dif cells and G40-Dif cells grown on collagen type I were treated with indicated concentrations of DIS and CBX. (**a**, **d**) Cell numbers were determined by the use of Casy1 cell counter. Mean and SEM are depicted of three replicates of triple measurements. (**b**, **e**) DNA fragmentation was analysed via flow cytometry of propidium iodide-stained nuclei. Mean and SEM are depicted of three independently conducted experiments in triplicate. (**c**, **f**) Cell movement was examined 24-56 hours post seeding and treatment of the cells with DIS and CBX. Cell velocity was examined in 8-hour intervals (n=20). Mean of experiments performed in duplicate are shown. Two-way ANOVA followed by Bonferroni's post-test was used to determine statistical significance (*p<0.05, **p<0.01, ***p<0.001).

Supp. Fig. 9

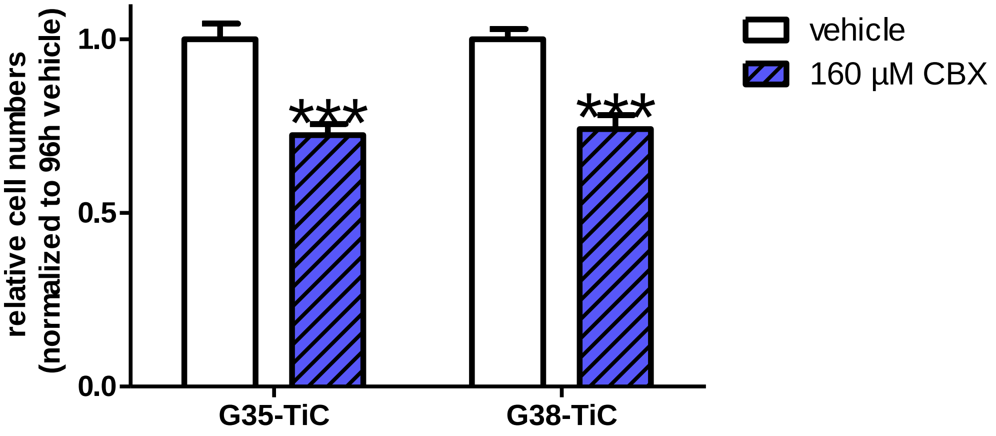

Supplementary Figure S9: The effect of CBX on cell count of two additional TiC populations.

G35-TiC and G38-TiC cells were either treated with water (vehicle) or with 160µM of Carbenoxolone (CBX). The cell number was measured after 96h and untreated controls were defined as 1. Mean and SEM of four independently conducted experiments in triplicate are depicted. Two-way ANOVA followed by Bonferroni's post-test was used to determine statistical significance (***$p<0.001$).

Supp. Fig. 10

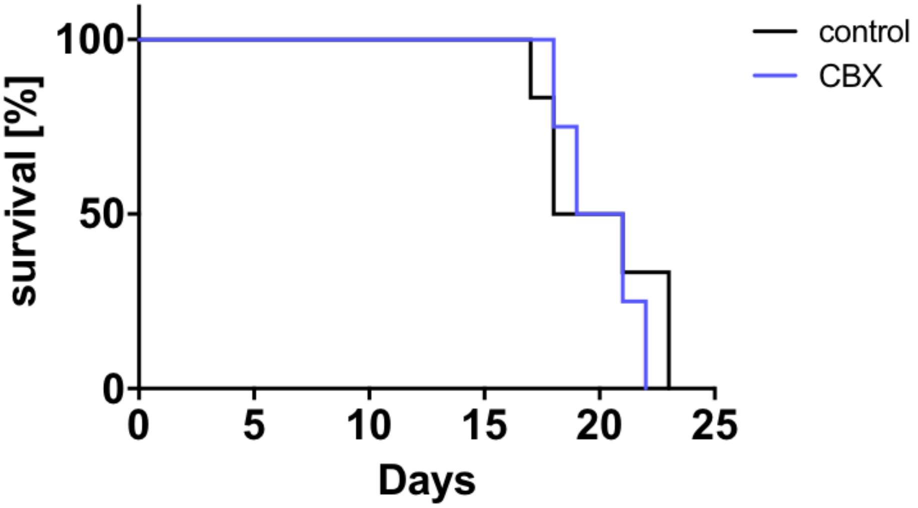

Supplementary Figure S10: Comparing the treatment effect CBX on tumour progression in an orthotrophic mouse model.

G35-TiCs were injected into a mouse brain and either left untreated or treated on Day 7 and 11 post-injection with 10mg/kg CBX/ INI-0602. Mice were sacrificed upon first symptoms; no statistically significant difference exists between the two groups (n=8).

Supplementary Table S1

DNA fragmentation [%]	G40-TiC in suspension	G40-Tic on collagen	Significance
vehicle	1.63	-0.80	ns
1µM DIS	2.44	-1.19	ns
40µM DIS	14.57	40.90	**
160µM CBX	46.64	62.61	ns
1µM DIS + 160µM CBX	48.89	76.28	**
40µM DIS + 160 µM CBX	23.18	50.97	***

Specific apoptosis in DIS- and CBX-treated G40-TiCs cultured in suspension in comparison to G40-TiCs cultured on collagen I.

After seeding, cells were treated immediately for 96hrs with 1 µM DIS, 40 µM DIS, 160 µM CBX or a combination of DIS and CBX. DNA fragmentation was analysed via flow cytometry of propidium iodide-stained nuclei and depicted as the mean of three independent experiments. Two-way ANOVA followed by Bonferroni's post-test was used to determine statistical significance (ns not significant, *$p<0.05$, **$p<0.01$, ***$p<0.001$).

Online supplementary files:

Movies S1-S4 show time-lapse videos of G40-Dif cells left untreated (S1), treated with DIS (S2), CBX (S3) or a combination thereof (S4). Videos comprise still images taken every ten minutes for 48 hours. Exemplary pictures taken from these files are shown in Figure 4.
